# Ouabain and chloroquine trigger senolysis of BRAF‐V600E‐induced senescent cells by targeting autophagy

**DOI:** 10.1111/acel.13447

**Published:** 2021-08-06

**Authors:** Valentin L’Hôte, Régis Courbeyrette, Guillaume Pinna, Jean‐Christophe Cintrat, Gwenaëlle Le Pavec, Agnès Delaunay‐Moisan, Carl Mann, Jean‐Yves Thuret

**Affiliations:** ^1^ Université Paris‐Saclay CEA CNRS Institute for Integrative Biology of the Cell (I2BC) Gif‐sur‐Yvette Cedex France; ^2^ Université Paris‐Saclay CEA INRAE Département Médicaments et Technologies pour la Santé (DMTS) SCBM Gif‐sur‐Yvette France

**Keywords:** cardioglycosides, cellular senescence, endoplasmic reticulum stress, melanoma, Na,K‐ATPase, senolytic, Src

## Abstract

The expression of BRAF‐V600E triggers oncogene‐induced senescence in normal cells and is implicated in the development of several cancers including melanoma. Here, we report that cardioglycosides such as ouabain are potent senolytics in BRAF senescence. Sensitization by ATP1A1 knockdown and protection by supplemental potassium showed that senolysis by ouabain was mediated by the Na,K‐ATPase pump. Both ion transport inhibition and signal transduction result from cardioglycosides binding to Na,K‐ATPase. An inhibitor of the pump that does not trigger signaling was not senolytic despite blocking ion transport, demonstrating that signal transduction is required for senolysis. Ouabain triggered the activation of Src, p38, Akt, and Erk in BRAF‐senescent cells, and signaling inhibitors prevented cell death. The expression of BRAF‐V600E increased ER stress and autophagy in BRAF‐senescent cells and sensitized the cell to senolysis by ouabain. Ouabain inhibited autophagy flux, which was restored by signaling inhibitors. Consequently, we identified autophagy inhibitor chloroquine as a novel senolytic in BRAF senescence based on the mode of action of cardioglycosides. Our work underlies the interest of characterizing the mechanisms of senolytics to discover novel compounds and identifies the endoplasmic reticulum stress‐autophagy tandem as a new vulnerability in BRAF senescence that can be exploited for the development of further senolytic strategies.

## Graphical abstract

Cardioglycosides are particularly potent senolytics in BRAF‐V600E‐induced senescence. Senolysis by cardioglycosides is not mediated by Na,K‐ATPase pump inhibition but rather by signal transduction. BRAF‐V600E‐induced senescent cells rely on increased autophagy flux for survival. Cardioglycosides decrease autophagy flux, thus killing BRAF‐V600E‐induced senescent cells with high potency. Accordingly, autophagy inhibitor chloroquine is also senolytic in BRAF‐V600E senescence.

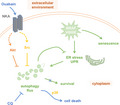

## 1 INTRODUCTION

Cellular senescence is recognized as a driving factor in an increasing number of diseases and disorders and the accumulation of senescent cells in tissues is an important driver of aging (Van Deursen, 2014). Senescence is elicited in response to a variety of stresses such as DNA damage, telomere attrition, or oncogene expression. It encompasses a diversity of phenotypes that are characterized notably by a stable—theoretically irreversible—proliferative arrest and the boosted secretion of inflammatory factors (Hernandez‐Segura et al., 2018). Upon commitment to senescence, cells undergo profound epigenetic and transcriptional reprogramming that result in important physiological changes and the reliance on specific pathways for survival (Soto‐Gamez et al., 2019). The differences between normal and senescent cells allow for the selective targeting of the latter by pharmacological means. Over the last few years, the specific elimination of senescent cells with so‐called senolytic drugs has proven to be a ground‐breaking new tool that facilitates the study of the in vivo impact of senescent cells and holds promise in therapeutic contexts in which senescence plays a major role.

The first demonstration of drug‐mediated senolysis was in therapy‐induced senescent (TIS) mouse lymphomas, which are more prompt to commit apoptosis than their non‐senescent counterparts in response to the inhibition of autophagy and glucose metabolism (Dörr et al., 2013). The most studied senolytic treatment so far is the combination of dasatinib and quercetin, which in mice was shown to delay the onset of aging and to alleviate osteoporosis, idiopathic pulmonary fibrosis (IPF), and Alzheimer's disease (Farr et al., 2017; Musi et al., 2018; Schafer et al., 2017; Zhu et al., 2015). The senolytic catalog later grew to include notably BH3 mimetics (Yosef et al., 2016; Zhu et al., 2016), HSP90 inhibitors (Fuhrmann‐Stroissnigg et al., 2017), and cardioglycosides (Guerrero et al., 2019; Triana‐Martínez et al., 2019). Senolytics recently entered the clinic for pilot human trials in IPF and diabetic kidney disease (Hickson et al., 2019; Justice et al., 2019). The ongoing discovery and characterization of novel senolytics is yielding insights into the mechanisms of senescence and aging and drives the development of innovative therapies for age‐related diseases.

BRAF‐V600E is a hyperactive mutant form of the BRAF kinase whose expression can lead to oncogene‐induced senescence (OIS) in normal fibroblasts and melanocytes (Carvalho et al., 2019; Michaloglou et al., 2005). This mutation is the initiator event in the formation of nevi (moles). Melanocytes acquiring the mutation initially proliferate, allowing the nevus to form, before entering senescence. Furthermore, BRAF‐V600E is found in 50% of melanoma, and it is estimated that about a third of these cancers arise from a pre‐existing nevus (Pampena et al., 2017; Shain et al., 2015). Employing senolytics to eliminate BRAF‐senescent melanocytes within nevi in patients at risk of developing melanoma might then be an efficient strategy for preventing malignant transformation. We thus decided to screen the Prestwick chemical library for senolytics targeting BRAF‐induced senescence, and we identified cardioglycosides, including ouabain, as extremely potent senolytics in BRAF‐V600E‐senescent human fibroblasts.

Cardioglycosides have been used in human medicine to treat congestive heart failure and atrial arrhythmias since the end of the 18th century. They increase the output force of the heart and regulate its contractions (Prassas & Diamandis, 2008). They inhibit the sodium potassium ATPase pump (Na,K‐ATPase or NKA), which participates in membrane potential maintenance by importing K+ ions and exporting Na+ ions across the plasma membrane. The mechanisms by which cardioglycosides have beneficial effects in heart failure patients, however, remain a matter of controversy: cardioglycosides binding to NKA both inhibit ion transport and trigger signal transduction pathways (Askari, 2019). They also have other cellular targets (Campia et al., 2009; Wang et al., 2014). In this study, we demonstrated that senolysis of BRAF‐senescent cells by cardioglycosides was due to Na,K‐ATPase signaling rather than membrane potential disruption. Ouabain induced signaling through Src, Akt, p38, and Erk in BRAF‐senescent cells. Importantly, we showed that increased endoplasmic reticulum stress and autophagy flux induced by BRAF‐V600E expression was a prerequisite for senolysis by ouabain that depended on inhibition of cytoprotective autophagy. Accordingly, we identified autophagy inhibitor chloroquine as a novel senolytic drug in BRAF‐V600E‐induced senescence.

## 2 RESULTS

### 2.1 Identification of cardioglycosides as senolytics in BRAF‐senescent cells

We screened the Prestwick drug repositioning library for molecules that would kill WI‐38hTERT human fibroblasts induced in senescence by the expression of an activated form of the c‐RAF kinase that we previously characterized (Jeanblanc et al., 2012). We identified several cardioglycosides as potential senolytics (digoxin, ouabain, strophanthidin, proscillarin A; see Section 4), and we set out to investigate their effects on senescent cells.

**FIGURE 1:**
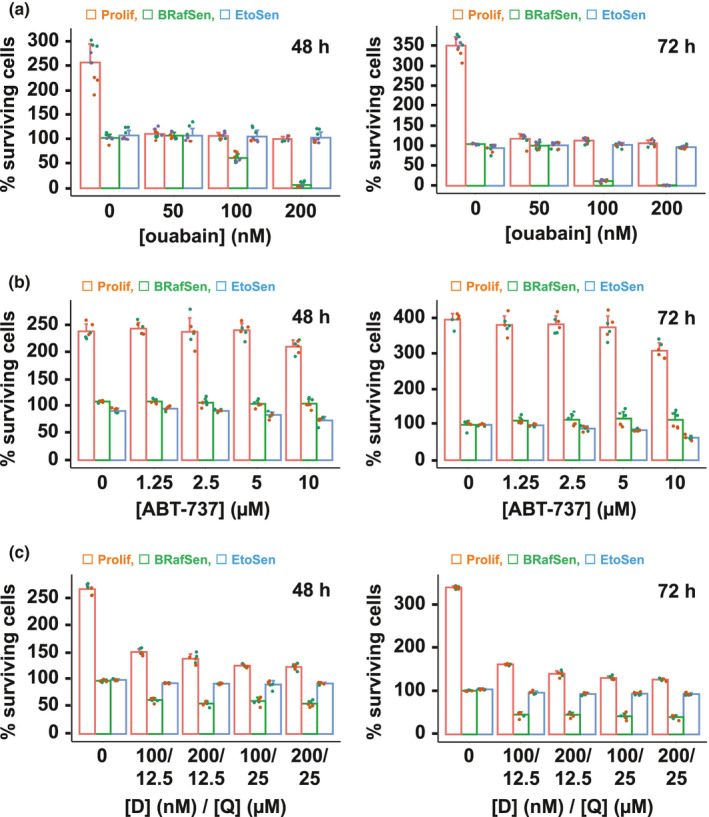
Ouabain is a potent senolytic in BRAF senescence. (a) Dose–response toxicity assay of cardioglycoside ouabain in BJ fibroblasts. Prolif: proliferating (red); BRafSen: BRAF‐V600E senescent (green); EtoSen: etoposide senescent (blue). Survival expressed as the percentage of viable cells remaining attached to the well after incubation with the drug for 48 h (left) or 72 h (right), normalized to the initial number of cells at the time of drug addition. Note that vehicle‐treated Prolif cells proliferated beyond 100%, whereas vehicle‐treated senescent cells neither proliferated nor died and thus remained at ~100%. Data were aggregated from three independent biological replicates. (b) Dose–response toxicity assay of BH3 mimetic ABT‐737 in BJ fibroblasts. Data were aggregated from two independent biological replicates. (c) Dose–response toxicity assay of the dasatinib (D) and quercetin (Q) combination in BJ fibroblasts. Data were aggregated from two independent biological replicates. For all panels, colored overlapping dots represent independent replicates

First, we assessed the senolytic potential of ouabain, and other cardioglycosides, on a human fibroblast BJ hTERT cell line that we recently described, in which senescence can be triggered by the inducible expression of BRAF‐V600E (Carvalho et al., 2019). In Figure 1a, we measured the toxicity of ouabain in proliferating cells (Prolif) versus cells rendered senescent by the expression of BRAF‐V600E (OIS, BRafSen) or by etoposide treatment (DNA damage senescence, EtoSen). Briefly, we induced senescence for 1 week, and we incubated the cells in 96‐well plates for 48 or 72 h with increasing concentrations of the drug. We counted surviving cells after fixation and nuclear staining as previously described (Carvalho et al., 2019). The graphs in Figure 1a show the percentage of cells counted after 48 and 72 h, relative to the initial number of cells at the time of drug addition (see Section 4). Toxicity on senescent cells is thus seen as a percentage of surviving cells below 100%. Remarkably, at concentrations where ouabain achieved near complete toxicity in BRafSen cells, EtoSen cells were not affected. At these concentrations, there was no increase in the number of proliferating cells (see for instance 100% surviving cells at 200 nM ouabain for 48 and 72 h), indicating that the drug was cytostatic, as previously described with cardioglycosides (Bloise et al., 2009). This proliferative arrest was partially reversible, as a fraction of the cells recovered the ability to carry out S‐phase (Figure S1A), or to grow into colonies (Figure S1B), upon withdrawal of the drug. In BRafSen cells, ouabain triggered apoptosis, as evidenced by PARP1 cleavage (Figure S2A), loss of mitochondrial potential and nuclear condensation (Figure S2B). Hence, at low concentrations, ouabain was a potent senolytic in BRafSen but not EtoSen cells, while inducing reversible cytostasis in proliferating cells. Two other cardioglycosides, digoxin and strophanthidin, behaved similarly (Figure S3A‐B).

We also compared the potential of ouabain to that of previously described senolytic drugs, namely navitoclax/ABT‐263 (Zhu et al., 2016), ABT‐737 (Yosef et al., 2016), alvespimycin/17‐DMAG (Fuhrmann‐Stroissnigg et al., 2017), dasatinib (Zhu et al., 2015), and the dasatinib and quercetin combination (D + Q, Zhu et al., 2015). As shown in Figure 1b–c, ABT‐737 exhibited a senolytic effect in EtoSen but not BRafSen cells, while D + Q was senolytic in BRafSen cells, albeit to a lesser extent than ouabain. Navitoclax was also senolytic only in EtoSen cells (Figure S4A). The other drugs were not senolytic in our models (Figure S4B‐C). Thus, in BJ cells, ouabain appeared to be more potent and specific of BRAF‐V600E‐induced senescence than any of the reference senolytics we tested. We therefore sought to decipher the mode of action of ouabain in BRafSen cells and to infer new senolytic drugs targeting the same cellular vulnerabilities.

### 2.2 Senolysis by cardioglycosides is mediated by the Na,K‐ATPase pump

**FIGURE 2:**
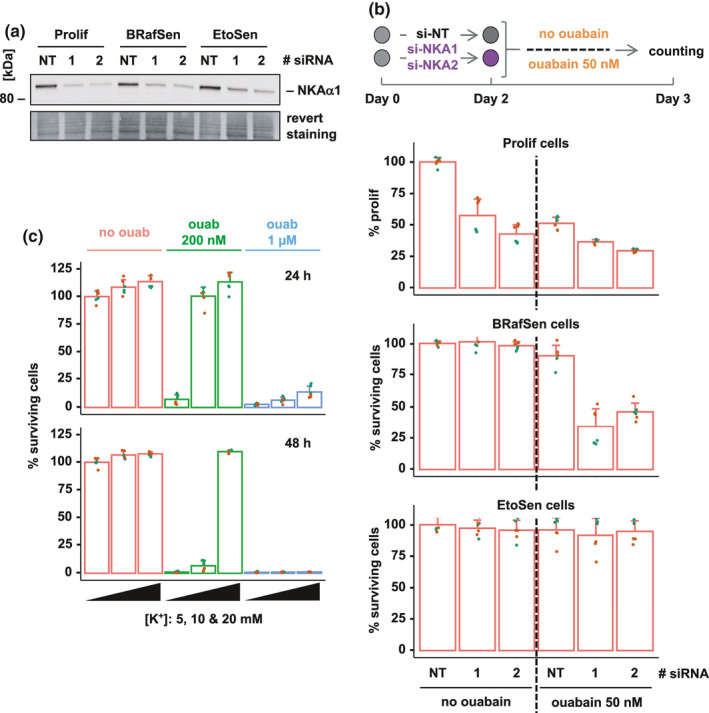
Involvement of the Na,K‐ATPase pump in senolysis by ouabain. (a) Protein levels of the Na,K‐ATPase pump subunit α1 (NKAα1) in BJ cells transfected for 48 h with a control no target siRNA (“NT”) or ATP1A1 (gene encoding NKAα1) siRNAs (si‐NKA1 “1” or si‐NKA2 “2”), assessed by Western blotting. Revert staining: total protein staining. Representative experiment of two independent biological replicates. (b) Toxicity and proliferation assay of 50 nM ouabain in Prolif, BRafSen, and EtoSen cells, following siRNA‐mediated ATP1A1 depletion. As illustrated in the top diagram, cells were transfected with the siRNAs for 48 h and then further incubated with 50 nM ouabain or vehicle for 48 h. For Prolif cells, cell number was normalized to the final number of proliferating cells set as 100% (transfection with control si‐NT, no ouabain). We observed no cell death in the wells. Data were aggregated from two independent biological replicates. (c) Protection of BRafSen cells from senolysis by KCl supplementation. Cells were treated for 24 h (top) or 48 h (bottom) with vehicle (red), 200 nM ouabain (green), or 1 µM ouabain (blue). Treatments performed in the presence of increasing K+ concentrations: 5 mM (left bar of a given color group), 10 mM (center bar of a given color group), 20 mM (right bar of a given color group). Data were aggregated from two independent biological replicates. For all panels, colored overlapping dots represent independent replicates

Ouabain and the other cardioglycosides isolated in our screening of the Prestwick library bind to the alpha subunit of the Na,K‐ATPase pump (NKA). NKA is composed of three subunits (α, β and FXYD‐like) with transmembrane domains (Clausen et al., 2017). In BJ cells, transcriptomic data that we previously obtained (Carvalho et al., 2019) indicated that of the four genes encoding α subunit isoforms, ATP1A1 was the most expressed (Figure S5A). We observed a modest increase in ATP1A1 expression in RT‐qPCR 7 days after senescence induction, the time at which cardioglycoside senolysis was assessed (Figure S5B). Accordingly, Western blotting did not reveal major changes in NKAα1 protein levels (encoded by ATP1A1) in senescence (Figure 2a). We depleted ATP1A1 mRNA using two different siRNAs, si‐NKA1 and si‐NKA2, in proliferating, BRafSen, and EtoSen BJ cells. Two days after siRNA treatment, NKAα1 levels were significantly reduced (Figure 2a and S5C). Depletion of ATP1A1 in itself (no ouabain) had no effect on the survival of BRafSen and EtoSen cells but slowed proliferation of non‐senescent BJ cells (Figure 2b). However, we observed that reducing NKAα1 levels sensitized BRafSen, but not EtoSen cells, to ouabain. 50 nM ouabain killed more than 50% of NKAα1‐depleted BRafSen cells in 24 h, with little toxicity in si‐NoTarget control BRafSen cells. The potentiation of ouabain by the depletion of NKAα1 in BRafSen cells strongly suggested that the drug acted by binding to the pump.

The Na,K‐ATPase pump actively transports Na+ and K+ ions against their respective electrochemical gradients across the plasma membrane, thus participating in transmembrane potential maintenance. Extracellular potassium is imported into the cell by Na,K‐ATPase. It competes with ouabain for binding to NKAα1 (Noël et al., 2018). In Figure 2c, BRafSen cells were incubated with 200 nM or 1 µM ouabain, with increasing concentrations of potassium. At 5 mM potassium (400 mg/L KCl, the normal concentration in culture media), ouabain killed nearly all BRafSen cells at both concentrations in 24 or 48 h. Doubling the concentration of potassium to 10 mM (800 mg/L KCl) completely protected the cells from 200 nM ouabain for 24 h, but not for 48 h. Although an even higher potassium concentration of 20 mM (1.4 g/L KCl) successfully protected the cells from 200 nM ouabain even for 48 h, increasing the concentration of ouabain to 1 µM overcame the protective effect of supplemental KCl. These results reflected the competition between ouabain and K+ ions for binding to the NKAα1 subunit as reported, hence confirming that the effect of ouabain on BrafSen cells was mediated by the Na,K‐ATPase. However, the toxicity of ouabain to BRafSen, but not EtoSen or Prolif cells, could not be explained by a decrease in ATP1A1 expression levels (Figure 2a).

### 2.3 Senolysis by cardioglycosides in BRAF‐senescent cells is not due to ion transport inhibition

**FIGURE 3:**
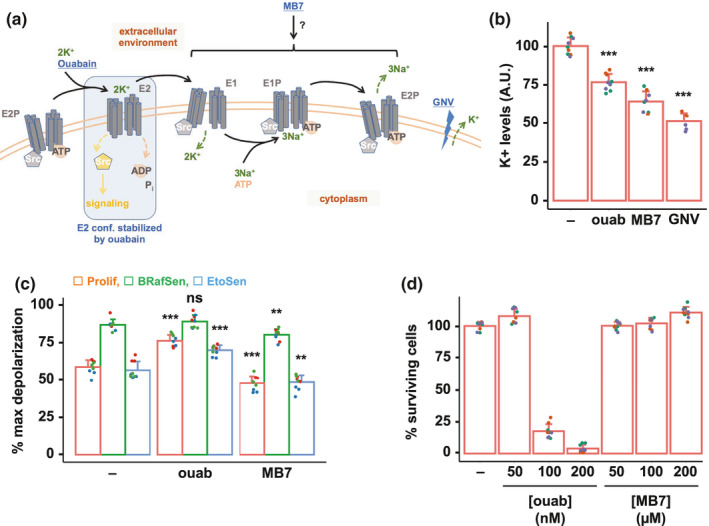
Senolysis by ouabain is not mediated by inhibition of ion transport. (a) Diagram of the Na,K‐ATPase's (NKA) Albers‐Post cycle. The various conformations adopted by the pump are indicated in gray. Compounds used in the study are in blue and underlined. In basal conditions, NKA binds Src and keeps it inactive. The NKA‐Src interaction is relieved when the pump adopts the E2 conformation, bound and stabilized by ouabain. GNV: gramicidin + nigericin + valinomycin. GNV is a combination of ionophores creating pores in the plasma membrane, resulting in an increased efflux of potassium from the intracellular to the extracellular medium. (b) Relative intracellular K+ levels of BRafSen cells after a 7‐h incubation with vehicle, 200 nM ouabain, or 200 µM MB7. GNV: positive control, treatment for 30 min with GNV 10 µM each. Following treatment with the compounds, cells were incubated with ION Potassium Green‐2 AM, a fluorescent potassium indicator. Fluorescence of live cells was measured in whole wells. The fluorescence of the indicator is proportional to intracellular K+ levels. Data were aggregated from three independent biological replicates. (c) Relative plasma membrane depolarization measurement in Prolif, BRafSen, and EtoSen cells after a 7‐h incubation with vehicle, 200 nM ouabain, or 200 µM MB7. Cells were incubated with 500 nM DiSBAC, whose fluorescence increases upon depolarization. Results were normalized to fluorescence intensity of cells in which maximum depolarization was achieved with 80 mM potassium gluconate. Data were aggregated from three independent biological replicates. (d) Dose–response toxicity assay of MB7 and ouabain in BRafSen cells. Cells were treated with increasing concentrations of MB7 and ouabain for 72 h. Note that 200 µM MB7 was not toxic in BRafSen cells despite inducing a similar K+ depletion as 200 nM ouabain, which conversely killed virtually all cells in 72 h. Data were aggregated from three independent biological replicates. For all panels, colored overlapping dots represent independent replicates. Results from statistical tests performed as described in the Section 4 are indicated as follows: *p < 0.05; **p < 0.01; ***p < 0.001

The molecular mechanism by which NKA functions as an ion pump is classically modeled as the Albers‐Post cycle depicted in Figure 3a, in which the pump adopts a series of different conformations (Cui & Xie, 2017). Besides its role as an ion transporter, the Na,K‐ATPase is also a receptor for signal transduction. NKAα1 notably interacts with Src, keeping it inactive in protein complexes. Upon binding, ouabain stabilizes Na,K‐ATPase in the E2 conformation, which both inhibits the ion transport function of the pump and releases Src (Figure 3a), resulting in the activation of various signaling pathways (Nie et al., 2020; Tian et al., 2006). Ouabain‐induced NKA signaling has been detected at concentrations that were too low to detect measurable changes in ion transport resulting from pump inhibition at the level of the cell (Prassas & Diamandis, 2008). Therefore, we next asked whether senolysis of BRafSen cells resulted from pump inhibition and/or Na,K‐ATPase signaling.

To distinguish between the two possibilities, we employed a distinct small molecule inhibitor of the Na,K‐ATPase pump which does not trigger signaling, MB7 (3,4,5,6‐tetrahydroxyxanthone). The affinity of MB7 for the various NKA conformations is not known. However, based on in vitro experiments, it was suggested that MB7 does not stabilize the E2 conformation (Zhang et al., 2010), and MB7 failed to trigger the activation of several signaling pathways reportedly triggered by ouabain binding to the Na,K‐ATPase (Cui & Xie, 2017). Inhibition of the pump by MB7 has been characterized in vitro with the purified protein, but never in cultured cells. NKA inhibition leads to a drop in intracellular K+ levels. Indeed, using a fluorescent potassium indicator (ION Potassium Green‐2 AM, see Section 4), we observed a similar decrease in the levels of cytoplasmic potassium upon treatment with 200 nM ouabain or 200 µM MB7 (Figure 3b). As a positive control, we used a combination of ionophores gramicidin, nigericin, and valinomycin to induce membrane pore formation and potassium efflux. Using the plasma membrane potential indicator DiSBAC2(3), whose fluorescence correlates with depolarization, we measured that the plasma membrane of BRafSen cells was constitutively depolarized compared to proliferating and EtoSen cells (Figure 3c). 200 nM ouabain induced a slight, non‐significant further depolarization of BRafSen cells, while treatment with 200 µM MB7 resulted in a slight membrane hyperpolarization. The effect of both drugs on membrane potential was more pronounced in proliferating and EtoSen cells whose plasma membranes were more polarized. Importantly, 200 µM MB7 was not toxic to BRafSen cells even if incubated for 72 h (Figure 3d), despite inhibiting potassium import to the same extent as 200 nM ouabain.

### 2.4 Cardioglycoside‐induced Na,K‐ATPase signaling is required for senolysis

**FIGURE 4:**
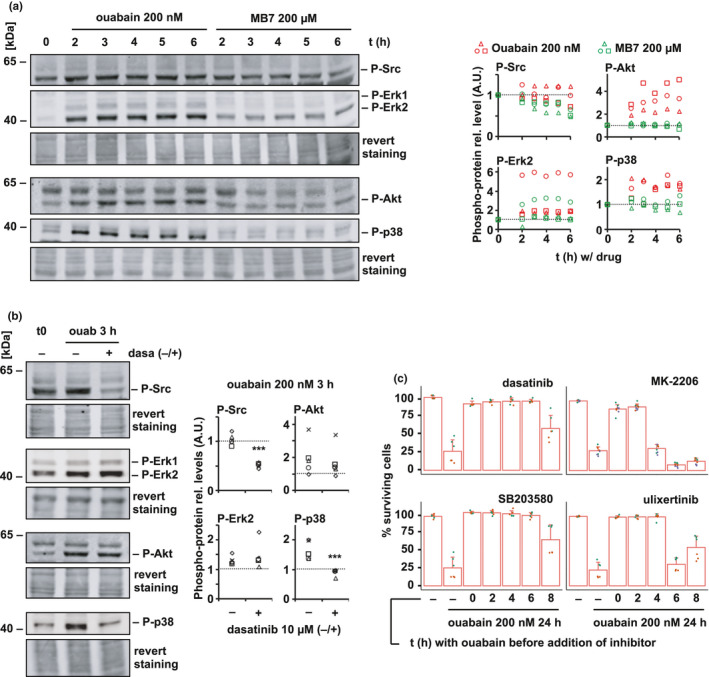
Senolysis by ouabain is mediated by Na,K‐ATPase signal transduction. (a) Phosphorylation of Src, Akt, p38, and Erk, in BRafSen cells treated with 200 nM ouabain or 200 µM MB7 in BRafSen cells, as assessed by Western blotting. Revert staining: total protein staining. Representative blot of three independent biological replicates. Quantified bands were normalized to revert staining within each lane then to t0. Shaped data points represent independent replicates. (b) Phosphorylation of Src, Akt, p38, and Erk, after 200 nM ouabain for 3 h in the presence or absence of Src inhibitor dasatinib. Revert staining: total protein staining. Representative blot of five independent biological replicates. Quantified bands were normalized to revert staining within each lane then to t0. (c) Time course rescue experiment of senolysis in BRafSen cells by signaling inhibitors of Src (10 µM dasatinib), p38 (25 µM SB203580), Akt (2 µM MK‐2206), and Erk (1 µM ulixertinib). Cells were treated with 200 nM ouabain, and inhibitors were added at various time points thereafter. Survival was assessed after 24 h incubation with ouabain. Data were aggregated from two independent biological replicates. For all panels, colored overlapping dots represent independent replicates. Results from statistical tests performed as described in the Section 4 section are indicated as follows: *p < 0.05; **p < 0.01; ***p < 0.001

Since ion transport inhibition appeared to be insufficient for the senolysis of BRafSen cells, we next investigated signaling pathways reportedly activated by cardioglycosides (Cui & Xie, 2017; Prassas & Diamandis, 2008). Ouabain, but not MB7, rapidly induced the phosphorylation of Akt (T308), p38 (T180/Y182), and Erk (T202/Y182; Figure 4a). Phosphorylation of Src (Y530) was already high in untreated BRafSen cells. Quantification showed that this phosphorylation was sustained by ouabain treatment, but MB7 decreased the abundance of phospho‐Src (Figure 4a). Immunoblots of total proteins are provided in Figure S6A.

A subset of cellular Src is located in signaling complexes with NKA, and its activation can often be the first step in ouabain‐induced signaling. Akt has also been described to be activated by cardioglycoside binding to NKA in a Src‐independent manner (Wu et al., 2013). Src inhibitor dasatinib decreased ouabain‐induced activation of Src and p38, but not Akt and Erk (Figure 4b). This suggested that cardioglycosides triggered the activation of a Src/ p38 axis in parallel to Src‐independent Akt and Erk axes. Immunoblots of total proteins are provided in Figure S6B.

To determine whether senolysis was actually dependent on the signaling triggered upon binding of cardioglycosides to NKA, we performed a time course rescue experiment with inhibitors. We treated BRafSen cells with ouabain, before introducing signaling inhibitors at various time points. We assessed cell survival 24 h after ouabain addition (Figure 4c). Dasatinib (Src inhibitor), MK‐2206 (Akt inhibitor), SB203580 (p38 inhibitor), and ulixertinib (Erk inhibitor) were effective in protecting BRafSen cells from senolysis if introduced in the first hours of incubation with ouabain. Their potency decreased with time, suggesting that the signaling events underlying cell death induction with 200 nM ouabain took place in the first 8 h of contact with the drug. The fact that inhibiting pathways associated with NKA transduction protected the cells from death was a further indication that senolysis of BRafSen cells by ouabain was primarily due to signaling rather than inhibition of ion transport, although we cannot exclude that a further slight depolarization of BRafSen cells by ouabain may participate in cell death.

### 2.5 BRAF‐V600E sets the stage for cardioglycoside senolysis

**FIGURE 5:**
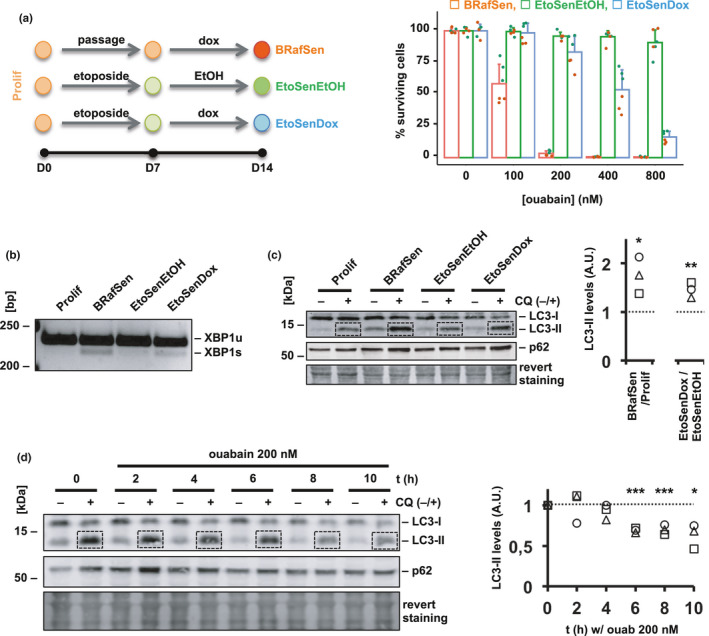
BRAF‐V600E expression induces ER stress and autophagy and sensitizes cells to ouabain. (a) Dose–response toxicity assay of ouabain in BJ cells with and without expression of BRAF‐V600E. As shown in the diagram, senescence was induced in BJ cells by incubation with etoposide (20 µM for 1 week, EtoSen cells). EtoSenEtOH cells (green): as a negative control, EtoSen cells were incubated with doxycycline vehicle (0.01% ethanol) for 1 week. EtoSenDox cells (blue): etoposide was withdrawn from EtoSen cells that were further incubated with 1 µg/ml doxycycline for 1 week to induce the expression of BRAF‐V600E. BRafSen cells (red): OIS in BJ cells was induced by incubating the cells with 1 µg/ml doxycycline for 1 week. Survival was assessed following a 48‐h incubation with increasing concentrations of ouabain. Data were aggregated from two independent biological replicates. (b) XBP1 mRNA splicing in Prolif, BRafSen, EtoSenEtOH, and EtoSenDox cells, assessed by RT‐PCR (reverse transcription coupled with polymerase chain reaction). Total RNA was extracted and reverse transcription was performed on an equal amount of RNA across all four cell types. XBP1 cDNA was then amplified by PCR, and PCR products loaded on a 3.5% agarose gel to separate the unspliced (u) and the spliced (s) XBP1 products. The detection of XBP1s is associated with endoplasmic reticulum (ER) stress. Representative experiment of two independent biological replicates. (c) Autophagy flux in Prolif, BRafSen, EtoSenEtOH, and EtoSenDox cells, assessed by Western blotting. Cells were treated with 50 µM chloroquine (CQ) or vehicle for 2 h prior to protein extraction. CQ blocks autophagosome degradation, thus allowing to assess the rate of autophagosome formation during the treatment 2‐h time window. Representative blot of three independent biological replicates. Quantified LC3‐II bands in CQ+ samples were normalized to revert staining within each lane then to control. (d) Effect of ouabain on autophagy flux in BRafSen cells, assessed by Western blotting. Cells were treated with 200 nM ouabain for increasing periods of time. 50 µM CQ or vehicle added during the last 2 h of incubation prior to protein extraction. Representative experiment of three independent biological replicates. Quantified LC3‐II bands in CQ+ samples were normalized to revert staining within each lane then to t0. For all panels, colored overlapping dots represent independent replicates. Results from statistical tests performed as described in the Section 4 are indicated as follows: *p < 0.05; **p < 0.01; ***p < 0.001

We then sought an explanation for the selectivity of ouabain for BRafSen cells over EtoSen and Prolif cells. To gain insight into the implication of BRAF‐V600E in the susceptibility to cardioglycosides, we induced expression of BRAF‐V600E with doxycycline for 1 week in EtoSen cells (EtoSenDox cells) and assessed their survival in the presence of ouabain. We compared their survival to control EtoSen cells treated with ethanol, the doxycycline vehicle (EtoSenEtOH). EtoSenEtOH cells were unaffected by ouabain within the concentration range tested, while EtoSenDox cells became sensitive to it, although to a lesser extent than BRafSen cells (Figure 5a). We concluded that BRAF‐V600E expression conferred susceptibility to senolysis by ouabain. Moreover, ouabain's toxicity did not depend on sustained expression of BRAF‐V600E in BRafSen cells and acute overexpression of BRAF‐V600E in proliferating cells failed to sensitize them to ouabain (Figure S7). Thus, senolysis by ouabain required the cells to be senescent or to express BRAF‐V600E for a prolonged time.

In melanoma cell lines, BRAF‐V600E was shown to induce chronic endoplasmic reticulum (ER) stress and subsequent upregulation of autophagy flux (Corazzari et al., 2015). It is also known that autophagy is important for the onset of senescence induced by BRAF‐V600E (Liu et al., 2014), but the status of ER stress and autophagy flux in cells once OIS is established has been less studied. The unfolded protein response (UPR) is one of the processes triggered as a consequence of ER stress and can thus be monitored as an indicator of ER stress magnitude. BRafSen cells exhibited a heightened rate of XBP1 mRNA splicing, indicative of constitutive UPR (Figure 5b). Interestingly, the splicing of XBP1 mRNA was also increased in EtoSenDox cells compared to EtoSenEtOH cells.

Variations in autophagy flux between conditions can be assessed by comparing the levels of autophagy proteins in these conditions when there is flux (CQ−) and when flux is blocked during a short period of time with chloroquine (CQ+), which inhibits autophagosome degradation (Klionsky et al., 2016). LC3 is a protein that is converted from its LC3‐I form to its LC3‐II form upon incorporation into autophagosomes and as such is one of the most used markers for autophagy. An increase in LC3‐II levels can result from an upregulation of autophagosome formation (stimulated flux) as well as from a blockade of autophagosome degradation (interrupted flux). An increase in LC3‐II levels between conditions when flux is blocked shortly with chloroquine is indicative of upregulated autophagy. Another common indicator of autophagy flux is p62, which mediates the recruitment of ubiquitinated substrates into autophagosomes, and whose degradation is primarily controlled through autophagy, though the correlation of p62 levels to autophagy flux is less straightforward than with LC3 (Klionsky et al., 2016). In BRafSen and EtoSenDox cells, the accumulation of LC3‐II in the presence of CQ was more important than in Prolif and EtoSenEtOH cells, respectively (Figure 5c). Hence, BRAF‐V600E expression resulted in augmented basal autophagy flux. To assess the relevance of autophagy in senolysis by cardioglycosides, we treated BRafSen cells with ouabain for various durations, introducing CQ in the last 2 h before protein extraction (Figure 5d). The accumulation of LC3‐II during CQ treatment declined over time with ouabain, indicating that ouabain decreased the rate of autophagosome formation and autophagy flux in BRafSen cells.

Collectively, these data showed that BRAF‐V600E expression resulted in chronic ER stress and increased autophagy flux, which was disrupted by ouabain treatment.

### 2.6 Autophagy inhibitors are senolytic in BRAF senescence

BRAF‐V600E‐induced autophagy in response to increased ER stress is often a cytoprotective process (Rather et al., 2020), and pharmacological inhibition of autophagy was shown to improve the potency of anti‐cancer drugs in melanoma harboring the BRAF‐V600E mutation (Goodall et al., 2014; Ma et al., 2014). We hypothesized that autophagy flux inhibition may underlie the specific senolysis of BRafSen cells by cardioglycosides and that, therefore, other autophagy inhibitors should behave similarly.

**FIGURE 6:**
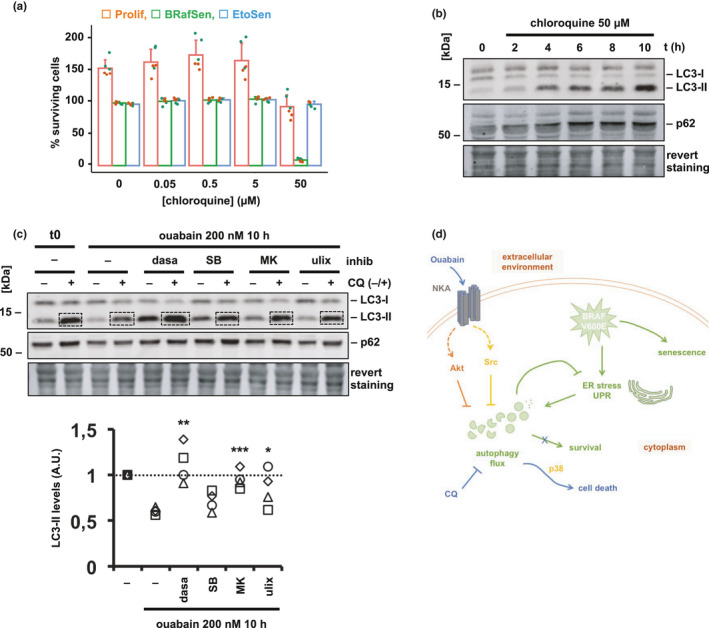
Autophagy inhibitor chloroquine is senolytic in BRAF senescence. (a) Dose–response toxicity assay of chloroquine in BJ cells. Cells were incubated with chloroquine for 24 h before survival assessment as described above. Data were aggregated from two independent biological replicates. (b) Effect of chloroquine on autophagy flux in BRafSen cells, assessed by Western blotting. Cells were treated with 50 µM chloroquine for increasing periods of time. Representative blot of two independent biological replicates. (c) Effect of signaling inhibitors on ouabain‐induced autophagy flux decrease in BRafSen cells, assessed by Western blotting. Cells were treated for 10 h with 200 nM ouabain in combination with an inhibitor of Src (10 µM dasatinib, dasa), p38 (25 µM SB203580, SB), Akt (2 µM MK‐2206, MK), or Erk (1 µM ulixertinib, ulix), or with vehicle. 50 µM CQ or vehicle added during the last 2 h of incubation, prior to protein extraction. Revert staining: total protein staining. Representative blot of four independent biological replicates. Quantified LC3‐II bands in CQ+ samples were normalized to revert staining within each lane then to t0. (d) Model of the senolysis of BRAF‐senescent cells by ouabain and chloroquine. BRAF‐V600E expression induces ER stress and a subsequent upregulation of autophagy flux, on which the cell depends for its survival. Ouabain triggers Na,K‐ATPase signaling resulting in autophagy inhibition, thus specifically killing BRafSen cells. Chloroquine, a blocker of autophagosome degradation, is also a senolytic in BRAF senescence. For all panels, colored overlapping dots represent independent replicates. Results from statistical tests performed as described in the Section 4 are indicated as follows: *p < 0.05; **p < 0.01; ***p < 0.001

We thus assayed the toxicity of autophagy inhibitors CQ and bafilomycin A1 (Baf A1) on proliferating, BRafSen and EtoSen BJ fibroblasts. Strikingly, these drugs were senolytic with toxicity profiles similar to that of cardioglycosides. 50 µM CQ killed virtually all BRafSen cells in 24 h, induced cytostasis in Prolif cells, and did not affect EtoSen cells (Figure 6a). Baf A1 achieved results of similar trend in 48 h, though with lesser potency (Figure S8). This resemblance in the selectivity of cardioglycosides and autophagy inhibitors for BRafSen cells further reinforced the hypothesis that ouabain senolysis was ultimately mediated by autophagy inhibition. CQ indeed inhibited autophagy flux in BRafSen cells, although at a different step than ouabain, as shown by the accumulation of LC3‐II and p62 due to blockade of autophagosome–lysosome fusion (Figure 6b).

To finally demonstrate that autophagy inhibition happened subsequently to cardioglycoside‐induced NKA signaling, we monitored autophagy flux in BRafSen cells treated with ouabain and signaling inhibitors. Autophagy flux was decreased by ouabain and significantly restored in the presence of Src or Akt inhibitors (Figure 6c). Inhibiting p38 did not significantly rescue the decrease in autophagy flux induced by ouabain. Inhibiting Erk partially restored autophagy flux, with lesser statistical significance.

Together, these data suggested that ER stress‐induced autophagy was the essential mechanism targeted in BRafSen cells during senolysis, inhibited either directly by CQ, or consequently to ouabain‐induced NKA signaling. CQ is a novel senolytic in BRAF senescence described herein for the first time.

## 3 DISCUSSION

Since Dörr and colleagues identified the first drugs in 2013, and Zhu and colleagues coined the term “senolytic” in 2015, the catalog of molecules identified as such keeps growing (Dörr et al., 2013; Zhu et al., 2015). Unfortunately, the molecular and cellular mechanisms underlying senolysis are often overlooked. Yet, determining the mode of action of a novel active compound and the series of events leading to cell death is likely to yield valuable information for the development of possibly even more potent drugs. We identified ouabain and other cardioglycosides during a screening for compounds modulating RAF‐induced senescence. Compared to reference senolytics, cardioglycosides appeared to be remarkably potent on BRAF senescence, which prompted us to try to decipher the mechanisms underlying their activity. During our investigation, two articles were concomitantly published introducing cardioglycosides as broad‐spectrum senolytics targeting a large range of senescent cells (Guerrero et al., 2019; Triana‐Martínez et al., 2019). BRAF senescence was not investigated in these studies. Here, we reported an important selectivity of ouabain for BRafSen cells in the 10–7 M range, but we also recently identified cardioglycosides as hit senolytic compounds in a new 20 µM screening of the Prestwick library including EtoSen cells (Figure S9A). In IMR90 cells induced in senescence by the expression of RasVal12 or by etoposide treatment, we measured a similar toxicity of cardioglycosides to what Guerrero et al. reported (Figure S9B‐C). Therefore, our data are in accordance with cardioglycosides being broad‐spectrum senolytics. Although they did not exclude a potential implication of other mechanisms, Guerrero and colleagues assigned senolysis by cardioglycosides to ion transport inhibition. Their investigation was albeit performed at 1 µM ouabain. They also reported ouabain‐mediated activation of Akt and p38. Triana‐Martínez and colleagues proposed a model in which the blocking of NKA by digoxin results in the inhibition of the Na,H‐exchanger, leading to a drop in pH and the death of senescent cells. Both studies interpreted the rescue of senolysis by supplemental KCl as intracellular K+ levels being replenished. Based on the analysis of the literature and our results, we suggest that this rescue, at least in BRafSen cells, is due to the competition between K+ and cardioglycosides for NKA binding. Depending on the molecule, the concentration, and the cell type considered, we do not exclude that senolysis by cardioglycosides might be mediated by ion transport inhibition. We propose however that our model is applicable to the remarkably potent senolysis of BRAF‐senescent cells by cardioglycosides.

Cardioglycosides primarily target the Na,K‐ATPase pump, even though they have been shown to interact with and regulate the activity of other proteins, such as SREBP2/SCAP (Sterol Regulatory Element‐Binding Protein‐2/SREBP Cleavage‐Activating Protein) and SRC‐3 (Steroid Receptor Coactivator‐3; Campia et al., 2009; Wang et al., 2014). Nevertheless here, depleting ATP1A1 potentiated the toxicity of ouabain in BRafSen cell, and complementing the culture medium with extra potassium protected the cells from apoptosis, indicating that NKA was indeed the target of cardioglycosides involved in senolysis. Although it is best known for transporting Na+ and K+ ions across the plasma membrane, NKA also has a well‐described role in signal transduction. In basal conditions, NKA physically interacts with and inhibits the tyrosine‐protein kinase Src within protein complexes. Src is released and activated when the Na,K‐ATPase pump is stabilized in the E2 conformation following cardioglycoside binding (Nie et al., 2020; Tian et al., 2006). Here, in BRafSen cells where NKAα1 was depleted, we observed a sensitization to ouabain‐induced apoptosis (Figure 2b). This was compatible with a role of NKA signaling, as an increase in basal Src activity has been described upon Na,K‐ATPase knockdown (Banerjee et al., 2018). MB7 (3,4,5,6‐tetrahydroxyxanthone) was developed to block the Na,K‐ATPase pumping function without activating signaling (Zhang et al., 2010). To our knowledge, inhibition of ion transport by MB7 had never been assayed in intact cells. Here, we demonstrated for the first time the ability of MB7 to inhibit the ion transport function of the Na,K‐ATPase in vivo. At concentrations where both compounds induced a similar inhibition of NKA‐mediated potassium import, ouabain was toxic to BRafSen cells while MB7 was not. On the other hand, resulting modulations of plasma membrane potential in BRafSen cells by ouabain were insignificant, while MB7 induced a slight hyperpolarization. Ouabain, but not MB7, rapidly induced signaling through Src, Akt, p38, and Erk. Moreover, inhibiting these pathways protected the cells from senolysis. These results indicated that the lethality of cardioglycosides on BRafSen cells was due to NKA signal transduction rather than inhibition of ion transport. However, BRafSen cells were constitutively much more depolarized than Prolif and EtoSen cells, which made it technically difficult to measure a significant further depolarization. Therefore, we cannot formally exclude that ouabain may further depolarize BRafSen cells and that this, through signaling induction, may play a role in cell death. Only a subset of Src is bound to NKA and activated by cardioglycosides. Given that phospho‐Src was already abundant in BRafSen cells, we assume that the activation of the NKA‐bound Src pool was probably not sufficient to see a significant increase in phospho‐Src abundance at the level of the cell. However, the fact that inhibiting Src protects cells from death, prevents p38 activation, and rescues autophagy flux shows that Src activity is indeed required for ouabain‐induced NKA signaling.

Autophagy and senescence share a variety of common inducing stressors. Oncogene activation leads to both autophagy and senescence, but the relationships between the two are yet to be fully understood, with reports of both positive and negative regulation of one by the other (Kwon et al., 2017). Autophagy mediates OIS onset, and its inhibition delays the proliferative arrest following oncogene expression (Young et al., 2009). TIS mouse lymphomas increase autophagy flux in response to proteotoxic stress caused by SASP production and are consequently more susceptible to bafilomycin A1‐induced apoptosis than their non‐senescent counterparts (Dörr et al., 2013). In melanoma, high levels of BRAF‐V600E concomitantly trigger a senescent‐like phenotype and autophagy through mTOR downregulation; inhibiting autophagy allowed cells to resume proliferation (Maddodi et al., 2010). Autophagy inhibitors can sensitize melanoma cells to chemotherapy (Goodall et al., 2014). Given that ouabain was remarkably potent in BRAF senescence and that it strongly activated Akt, upstream of mTOR, we hypothesized that cardioglycosides functioned as senolytics by disrupting autophagy, to which BRafSen cells would be more susceptible than proliferating and EtoSen cells. Senolysis by cardioglycosides was indeed dependent on BRAF‐V600E activation, which entailed increased ER stress and autophagy. The molecular mechanisms by which BRAF‐V600E leads to ER stress and autophagy in senescent cells are still to characterize. In melanoma, BRAF‐V600E‐induced ER stress can be mediated by the sequestration of ER chaperone GRP78 or the activation of p38 (Corazzari et al., 2015; Ma et al., 2014; Rather et al., 2020). Strikingly, autophagy inhibitor chloroquine exhibited the same toxicity profile as ouabain, killing virtually all BRafSen cells while not affecting EtoSen cells, and being cytostatic in proliferating cells. This strengthened the idea that autophagy was the fundamental process underlying the specific senolysis of BRafSen cells by cardioglycosides. We showed that distinct Src and Akt axes were implicated in the inhibition of autophagy flux by ouabain. p38, despite being activated downstream of Src, was not required for autophagy inhibition, and might thus be implicated in the subsequent steps leading to apoptosis following autophagy flux reduction. As such, the role of p38 in senolysis should be characterized in greater detail to better understand how inhibiting autophagy leads to BRafSen cell death. Although Erk appears to be required for senolysis, its implication in autophagy modulation remains partial, and its activation was not dependent on Src. Deciphering its precise role in the senolysis of BRafSen cells would however be complicated by the fact that it is a downstream target of BRAF‐V600E as well as being activated by cardioglycosides.

Taken together, our data suggest the following model for senolysis of BRAF‐senescent cells by ouabain (Figure 6d): the expression of BRAF‐V600E induces ER stress in BRafSen cells, in response to which autophagy flux is augmented and required for survival; ouabain binds to the Na,K‐ATPase pump, both inhibiting ion transport and triggering signal transduction, the latter being primarily relevant to senolysis; NKA‐mediated activation of Src and Akt leads to a decrease of autophagy flux. Due to BRAF‐V600E‐induced ER stress, a larger autophagy flux is required for the survival of BRafSen cells than that of Prolif and EtoSen cells. Consequently, ouabain preferentially induces apoptosis in senescent cells that express BRAF‐V600E, potentially through p38. Blocking autophagy flux at a different step such as autophagosome‐lysosome fusion using chloroquine, also results in senolysis.

Depending on the cell type and the inducing stressor, senescent cells rely on various survival mechanisms to resist apoptosis, such as ephrin signaling or changes in BCL2 family protein expression patterns (Soto‐Gamez et al., 2019; Yosef et al., 2016; Zhu et al., 2015). HSP90 inhibitors, azithromycin, fenofibrate, and quercetin‐functionalized MNPQ nanoparticles are senolytic drugs that all increase autophagy flux (Fuhrmann‐Stroissnigg et al., 2017; Lewinska et al., 2020; Nogueira‐Recalde et al., 2019; Ozsvari et al., 2018). We identify here cardioglycosides and chloroquine as senolytics that function by suppressing rather than inducing autophagy.

In conclusion, by deciphering the mechanisms of senolysis by cardioglycosides, and as previously shown in TIS (Dörr et al., 2013), we have identified autophagy as an essential process for the survival of BRAF‐senescent cells. The interplay between ER stress and autophagy and how it regulates the survival of BRAF‐senescent cells are yet to understand.

## 4 EXPERIMENTAL PROCEDURES

### 4.1 Materials

### 4.2 Screening of the Prestwick chemical library

### 4.3 Cell lines, cell culture, and senescence induction

### 4.4 Protein extraction and Western blotting

### 4.5 EdU incorporation

### 4.6 Clonogenicity assay

### 4.7 Fluorescence microscopy

### 4.8 Relative plasma membrane potential measurement

### 4.9 siRNA knockdown experiments

### 4.10 RNA extraction, RT‐qPCR, and RT‐PCR

See Supporting Experimental Procedures.

### 4.11 Dose–response toxicity assay

We seeded 4000 proliferating or 10,000 senescent cells per well in CostarAssay 96‐well plates (Corning 3904, Sigma‐Aldrich). The day after seeding, we treated cells with compounds of interest at various concentrations with at least three wells per condition (technical triplicates). In negative control wells, we treated cells with vehicle. In baseline control wells, we treated cells with 20 µM etoposide which very rapidly stops proliferation (Carvalho et al., 2019). The number of cells in these wells at the end of the incubation period was used as a surrogate for the initial cell number at the time of drugs addition and used for normalization. Cells were fixed and their nuclei stained by incubation in 1% paraformaldehyde (P6148, Sigma‐Aldrich), 0.1% Triton X‐100 (T8787, Sigma‐Aldrich), and 10 µg/ml Hoechst 33342 (B2261, Sigma‐Aldrich) in PBS, for 30 min at room temperature, then washed with PBS. Images were acquired on a CellInsight CX5 (Thermo Fisher Scientific) screening microscope with a 4× objective or on an Operetta (Perkin‐Elmer) screening microscope with a 10× objective. Surviving cell numbers in each well were determined by automated nucleus segmentation and counting.

### 4.12 Relative intracellular potassium content measurement

We seeded 10,000 BRafSen cells per well in 96‐well plates. 24 h after seeding, we applied various treatments to cells in triplicate wells. At the endpoint, we incubated cells with 5 µM ION Potassium Green‐2 AM K+ indicator (Abcam) for 40 min at 37°C. We withdrew the indicator and incubated the cells in fresh medium for 20 min at 37°C. Live cells were then imaged with a CellInsight CX5 microscope. We analyzed the total fluorescence of each well.

### 4.13 Statistical analyses

Statistical significance was assessed for all datasets by performing a bilateral unpaired Student's *t* test assuming equal variances, except for rescue experiments in which we performed a unilateral unpaired Student's *t* test assuming equal variances. A *p*‐value < 0.05 was considered significant. For intracellular potassium levels and membrane potential measurements, treated cells were compared to vehicle. For time course Western blotting experiments, quantified bands were compared to t0. For other Western blotting experiments, quantified bands were compared to control.

## CONFLICT OF INTEREST

The authors declare that they have no conflict of interest.

## AUTHOR’S CONTRIBUTIONS

VL, RC, GP, and JYT, performed the experiments. GP designed the Prestwick library screening strategy. JC prepared the Prestwick library for screening. GLP and ADM optimized XBP1 splicing analysis. CM and JYT conceptualized and supervised the study, and secured funding. VL, ADM, and JYT, analyzed the data. VL and JYT wrote the manuscript. All authors reviewed, edited, and validated the manuscript.

## Supporting information

Supinfo S1

## Data Availability

The data that support the findings of this study are available from the corresponding author upon reasonable request.
