# Supplementary material for: Ouabain and chloroquine trigger senolysis of BRAF‐V600E‐induced senescent cells by targeting autophagy
Source: Aging Cell. 2021 Aug 6;20(9):e13447. doi: 10.1111/acel.13447 (PMC8564827; doi:10.1111/acel.13447)
Supplement: Supplementary file 1 — Supinfo S1 [file ACEL-20-e13447-s001.pdf]

L'Hôte et al.

Supporting information

Supporting experimental procedures

Materials

The drug repositioning library was purchased from Prestwick Chemical (Illkirch, France). The following drugs were used in the study: doxycycline (D3447, Sigma-Aldrich), etoposide (E1383, Sigma-Aldrich), ouabain (O3125, Sigma-Aldrich), digoxin (D6003, Sigma-Aldrich), strophanthidin (S6626, Sigma-Aldrich), ABT-737 (S1002, Selleckchem), navitoclax / ABT-263 (S1001, Selleckchem), dasatinib (D074, TOKU-E), quercetin (Q0125, Sigma-Aldrich), alvespimycin / 17-DMAG (S1142, Selleckchem), MB7 / 3,4,5,6-tetrahydroxyxanthone (T5955, Sigma-Aldrich), ulixertinib (S7854, Selleckchem), SB203580 (S1076, Selleckchem), MK-2206 (S1078, Selleckchem), chloroquine diphosphate (C6628, Sigma-Aldrich), bafilomycin A1 (B1793, Sigma-Aldrich), 4-hydroxytamoxifen (H6278, Sigma-Aldrich), valinomycin (V0627, Sigma-Aldrich), gramicidin (C5502, Sigma-Aldrich), nigericin (481990, Merck Millipore).

Screening of the Prestwick chemical library

WI-38-hTERT/C-BRAF-ER cells in which senescence was induced with 20 nM 4-hydroxytamoxifen (Jeanblanc et al. 2012) were incubated with 1,030 molecules of the Prestwick drug repositioning library. The final drug concentration was 35 µM in 0.35% DMSO. Cells were incubated with drugs for 4 days before cell number was evaluated as described in Carvalho et al. (Carvalho et al. 2019). The primary screen was carried out in duplicate plates. For each plate, the mean number of cells and the corresponding standard deviation were calculated in 8 negative control wells (DMSO only). From this, we calculated the coefficient of variation for the negative control (CV, standard deviation as a percentage of the mean), and the plate was considered valid only if the CV was less than 5%. For every well, we calculated a score corresponding to the number of cells in the well minus the mean number of cells of the negative controls, divided by the standard deviation of the negative controls. For each molecule, we thus had two z-scores, corresponding to the difference in the number of cells with the molecule assayed and in the negative control, expressed as the number of standard deviations of the negative control. A molecule was determined a hit if a decrease in the number of cells was measured, with scores higher than 3 in both replicates. Of the 1,030 molecules assayed, 78 (8%) were positive. Of these hits, 25 molecules killed more than 50% of the cells. The 78 molecules were assayed at 20 µM and 8 µM, again on C-BRAF-ER-senescent

WI-38-hTERT cells and on quiescent WI-38-hTERT cells (kept in medium with 1% fetal bovine serum). We further selected molecules with senolytic potential when their lethality score compared to the negative control was 3 times higher in senescent cells compared to the quiescent cells (ΔZ = Zsenescent – Zquiescent > 3). 7 molecules were retained as bona fide candidates at 20 µM, among which digoxin (ΔZ=4.8), strophantineoctahydrate (ΔZ=4.8), stropanthidin (ΔZ=4.7), digoxigenin (ΔZ=4.3), proscillaridin A (ΔZ=4.5) and digitoxigenin (ΔZ=3.5). 4 passed at 8 µM, strophanthidin (ΔZ=3.8), digoxin (ΔZ=3.2), strophantine octahydrate (ΔZ=3.2) and proscillaridin A (ΔZ=3.2). We used ouabain (strophantine octahydrate) for further experiments to characterize the mechanisms of senolysis by glycosides.

Cell lines, cell culture and senescence induction

The BJ1-hTERT cell line was purchased from Clontech, and the BJ1-hTERT/pTRIPz-3HA-BRAF-V600E (BJ) cell line was generated as described in (Carvalho et al. 2019). Cells were cultured in MEM (Gibco #51200, Thermo Fisher Scientific) with FBS 9% (Eurobio), Glutamax 2 mM (A12860, Gibco), MEM non-essential amino acids 1X (M7145, Sigma-Aldrich), sodium pyruvate 1 mM (S8636), and penicillin-streptomycin 1X (P0781, Sigma-Aldrich), at 37°C and 5% CO_2 in ambient oxygen. Routine culture included splitting cells before confluence and regularly checking for mycoplasma infection. For oncogene-induced senescence (BRafSen), BRAF-V600E overexpression was triggered by incubating the cells with 1 µg/mL doxycycline for one week, renewing medium with doxycycline every 3 to 4 days. DNA damage senescence (EtoSen) was induced by incubating the cells with 20 µM etoposide for one week, renewing medium with etoposide every 3 to 4 days. Etoposide was withdrawn for subsequent experiments.

The IMR90/ER-RasVal12 cell line was a kind gift from Masashi Narita (Cambridge Research Institute, CRUK, Cambridge, UK), and the immortalized IMR90-hTERT/ER-RasVal12 cell line was generated as described in (Jeanblanc et al. 2012). Cells were cultured in high glucose DMEM (Gibco D6429, Thermo Fisher Scientific) with FBS 9% (Eurobio), at 37°C, 5% CO_2 and 5% oxygen. Routine culture included splitting cells before confluence and regularly checking for mycoplasma infection. For oncogene-induced senescence (RasVSen), RasVal12 overexpression was triggered by incubating the cells with 100 ng/mL 4-hydroxytamoxifen for one week, renewing medium with 4-hydroxytamoxifen every 3 to 4 days. DNA damage

senescence (EtoSen) was induced by incubating the cells with 50 µM etoposide for 48 h, then withdrawing the drug for 5 days before starting experiments.

Protein extraction and western blotting

We scraped cells on ice in culture media, collected and centrifuged them at 1000 g for 5 min at 4°C. We resuspended cells in ice-cold PBS and centrifuged them again at 1000 g for 5 min at 4°C. We carefully eliminated supernatant, and we resuspended cells in 2 volumes of PBS with protease and phosphatase inhibitors (B14001 and B15001A/B, Bimake) and 1 volume of 3X Sample Buffer. Final 1X Sample Buffer composition was: 250 mM Tris (T1503, Merck), 70 mM sodium dodecylsulfate (SDS) (1066934, Gibco), 0.4 mM ethylenediaminetetraacetic acid (EDTA) (8418, Merck), 1 M glycerol (24388.320, VWR Chemicals), 0.015% Serva Electrophoresis Serva Blue G (3505002, Thermo Fisher Scientific), 2.5% 2-mercaptoethanol (805740, Merck). We incubated samples at 70°C for 10 min, briefly sonicated them until viscosity was broken, and loaded them onto Bis-Tris (B7535, Sigma) acrylamide (A7802, Sigma) gels for separation by electrophoresis. We transferred proteins onto Amersham Protan 0.2 µm nitrocellulose blotting membranes (10600001, GE Healthcare Life Sciences). We blocked membranes in Intercept PBS Blocking Buffer (927-70001, Li-Cor) diluted ½ in PBS, or in Odyssey TBS Blocking Buffer (927-50000, Li-Cor) diluted ½ in TBS, for 1 h at room temperature with gentle shaking. We then incubated membranes overnight at 4°C with primary antibodies in blocking solution. We washed membranes in PBS or TBS with 0.1% Tween-20, and incubated them with NIR-secondary antibodies (Li-Cor) diluted in 0.1% Tween-20 PBS or TBS. We washed membranes in 0.1% Tween-20 PBS or TBS, and imaged them on a Li-Cor Odyssey CLx scanner. For detection of NKAα1, cells were harvested as described above, then resuspended in an appropriate volume of RIPA buffer with protease and phosphatase inhibitors. After a 30-min incubation on ice, we centrifuged samples at 17000 g for 15 min at 4°C. The supernatant was collected and the required volume of 3X Sample Buffer was added. Samples were incubated at 37°C for 15 min, then loaded on Bis-tris acrylamide gels for electrophoresis. Western blotting was performed as above.

For signaling and autophagy studies, cells were flash-frozen after washing with PBS. PBS was removed, and plates plunged in a dry ice ethanol bath for 1 min (cells were not in contact with ethanol). Plates were incubated at -80°C overnight then proteins were extracted by scraping cells on ice in 1X Sample Buffer containing protease and phosphatase inhibitors. Samples were

incubated at 70°C for 10 min and sonicated until viscosity was broken. We loaded proteins onto gels and carried out western blotting as above. See table S1 for the list of antibodies and their dilutions.

Secondary antibodies were IRDye Goat anti-mouse / anti-rabbit IgG (H+L) 680RD / 800CW (Thermo Fisher Scientific).

EdU incorporation

We seeded 4000 proliferating cells per well in 96-well plates and incubated them with 25 nM EdU (Molecular Probes, Invitrogen, Thermo Fisher Scientific) for 48 h. Cells were fixed with 1% formaldehyde (F1635, Sigma-Aldrich) for 15 min at room temperature. We detected incorporated EdU by click chemistry using the Click-iT EdU Imaging kit (Molecular Probes, Invitrogen, Thermo Fisher Scientific) following the manufacturer's instructions. Images were acquired on a CellInsight CX5 microscope. The percentage of EdU-positive cells in each well was determined after automated nucleus segmentation as described above.

Clonogenicity assay

We seeded 1000 cells per well in a 6-well plate (Corning 353046, Sigma Aldrich) and cultured for two weeks, renewing medium every 3 to 4 days. We used 20 µM etoposide as a negative control to block clone formation. Cells fixed with 2% formaldehyde for 10 min at room temperature, washed once with PBS and incubated with 0.1% crystal violet for 1 h at room temperature. Wells were washed 4x with PBS, and images acquired with a LiCor Odyssey CLx scanner at 700 nm.

Fluorescence microscopy

Cells were seeded in 96-well plates and treated with compounds of interest. Live cells were treated with 200 nM MitoTracker Red CMXRos (M7512, Invitrogen, ThermoFisher Scientific) for 15 min before fixation according to the manufacturer's protocol. Plates fixed with 1% formaldehyde for 15 min at room temperature. Cells permeabilized with 0.5% Triton X-100 for 10 min at room temperature. Blocking with 5% bovine serum albumin (BSA) (A7906, Sigma-Aldrich) and 0.1% Tween-20 (P1379, Sigma-Aldrich) in PBS, 1 h at room temperature. Nuclei stained with 500 ng/mL DAPI (D9542, Sigma-Aldrich) for 10 min at room temperature. Images acquired on a CellInsight CX5 microscope.

Relative plasma membrane potential measurement

We seeded 10000 cells per well in 96-well plates in serum-free MEM containing 500 nM DiSBAC2(3) (Thermo Fisher Scientific) and the various drugs to assess. The plates were incubated for 7 h. 10 µg/mL Hoechst 33342 was added to the cells 30 min before imaging. 10 min before imaging, maximum depolarization was achieved by adding 80 mM potassium D-gluconate (Sigma-Aldrich) in half the wells for each treatment. We imaged cells live with an Operetta screening microscope with a 10X objective. We calculated in each well the mean integrated DiSBAC fluorescence intensity per cell. Within each treatment, this value was normalized to the mean integrated DiSBAC fluorescence intensity in totally depolarized cells with potassium gluconate.

siRNA knock-down experiments

We incubated siRNAs (Ambion Life Technologies, Thermo Fisher Scientific) and Lipofectamine RNAiMAX Reagent (Invitrogen, Thermo Fisher Scientific) in Opti-MEM (Gibco #31985070, Thermo Fisher Scientific) for 25 min at room temperature. Cells were added to the siRNA mix then seeded in 96-well plates (5000 cells/well), or 6-well plates (250000 cells/well). Final reagent concentrations were 15 nM siRNA, 0.1% Lipofectamine RNAiMAX, and 25% OptiMEM. Target knock-down was assessed 48 hrours later by RT-qPCR and/or western blotting. Further treatments were applied as described. Surviving cells in 96-well plates were fixed and counted as described. ATP1A1 siRNAs were purchased from Ambion/Life Technologies (si-NKA1: s1719; si-NKA2: s1720).

RNA extraction, RT-qPCR, and RT-PCR

Cells were scraped on ice in culture media, collected, centrifuged at 1000 g for 5 min at 4°C, resuspended in ice-cold PBS and centrifuged again. Supernatant was eliminated and RNA extraction was done using the NucleoSpin RNA Plus kit (Macherey-Nagel, Hoerdt, France) following the manufacturer's protocol. RNA was eluted in ddH2O and quantified with a NanoDrop 2000 spectrophotometer (Thermo Fisher Scientific).

For ATP1A1 RT-qPCR, reverse transcription was done on 500 ng RNA with 10.2 ng/µL random hexamer primers (Invitrogen, Thermo Fisher Scientific), 250 µM dNTP, and 4 U/µL Maxima Reverse Transcriptase (Thermo Fisher Scientific) in M-MLV Reverse Transcriptase buffer (Thermo Fisher Scientific) with 10 min at 25°C, 30 min at 50°C, 5 min at 85°C. The cDNA was

diluted 1/5 in ddH2O and qPCR performed with a Luminaris Color HiGreen qPCR Master Mix (Thermo Fisher Scientific) with 300 nM primers, monitored on an IQ5 apparatus (Bio-Rad). Thermal cycling was: 2 min at 50°C, 10 min at 95°C; 45 cycles of 15 s at 95°C and 1 min at 60°C. GAPDH was used for normalization. qPCR primers were synthesized by Sigma-Aldrich. qPCR primer sequences were: ATP1A1 forward: 5'-GGTCCCAACGCCCTCACTC-3'; ATP1A1 reverse: 5'-ACCACACCCAGGTACAGATTATCG-3'; GAPDH forward: 5'-ATGGGGAAGGTGAAGGTCG-3'; GAPDH reverse: 5'-GGGGTCATTGATGGCAACAATA-3'.

For XBP1 RT-PCR, reverse transcription was done on 500 ng RNA with 1.25 µM oligo-dT (Sigma-Aldrich), 500 µM dNTP, 10 mM dithiothreithol (DTT) (Thermo Fisher Scientific), and 5 U/µL M-MLV Reverse Transcriptase (Thermo Fisher Scientific) in M-MLV Reverse Transcriptase buffer (Thermo Fisher Scientific) with 10 min at 70°C, 2.5 min at 62°C, 90 min at 42°C, 10 min at 95°C. We added DTT and M-MLV reverse transcriptase only during the 42°C step. We diluted cDNA 1/2 in ddH2O and then 1/5 in PCR reaction buffer. We performed PCR with 300 nM of each XBP1 primer, 125 µM dNTP (Thermo Fisher Scientific), 1.2 mM MgCl2 (Thermo Fisher Scientific), and 50 mU/µL Taq polymerase (Thermo Fisher Scientific) in MgCl2 Buffer (Thermo Fisher Scientific). Thermal cycling was: 4 min at 94°C; 35 cycles of 30 s at 94°C, 30 s at 60°C, and 50 s at 72°C; then 7 min at 72°C. PCR primers were synthesized by Sigma-Aldrich. PCR primer sequences were: XBP1 forward: 5'-GGAACAGCAAGTGGTAGA-3'; XBP1 reverse: 5'-CTGGAGGGGTGACAAC-3'. We loaded PCR products on a 3.5% agarose gel with 0.00005% ethidium bromide (Gen-Apex) for electrophoresis. We imaged the gel with a Fusion Solo S apparatus (Vilber).

Supporting Table 1: list of antibodies used in the study.

Protein target	Manufacturer	Reference	Dilution
NKAα1	Santa Cruz	sc-21712	1/500
SRC	Proteintech	60315	1/10000
phospho-SRC	Santa Cruz	sc-166860	1/200
AKT	Cell Signaling Technology	2920S	1/1000
phospho-AKT	Cell Signaling Technology	4060P	1/500
ERK	Cell Signaling Technology	4695	1/1000
phospho-ERK	Cell Signaling Technology	9106S	1/1000
p38	Cell Signaling Technology	9218	1/1000
phospho-p38	Cell Signaling Technology	9216	1/500
LC3	Sigma-Aldrich	L8918	1/500
p62	Sigma-Aldrich	P0067	1/1000
PARP1	Santa Cruz	sc-7150	1/1000

Figure S1. Reversibility of ouabain-induced cytostaticity in Prolif cells.
L'Hôte et al.

A

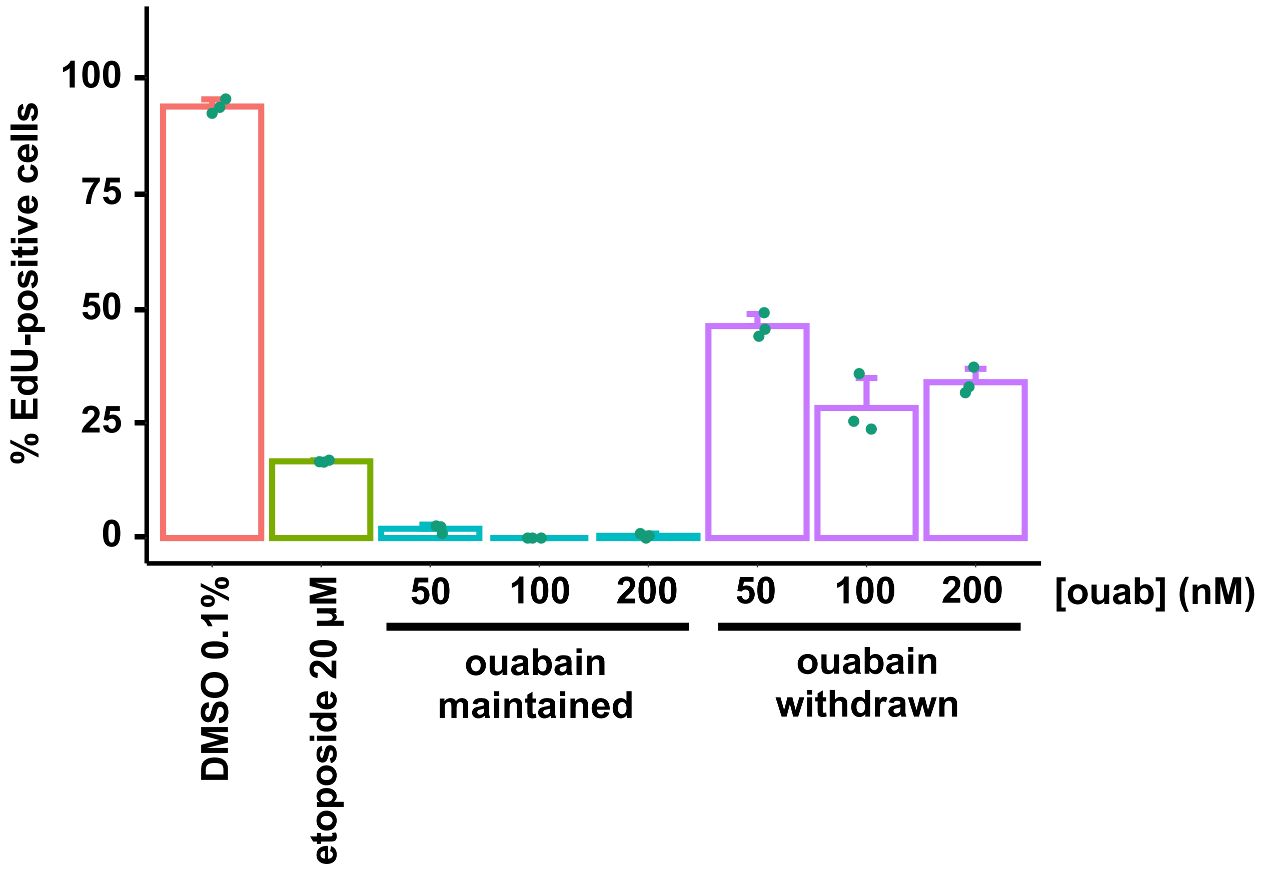

B

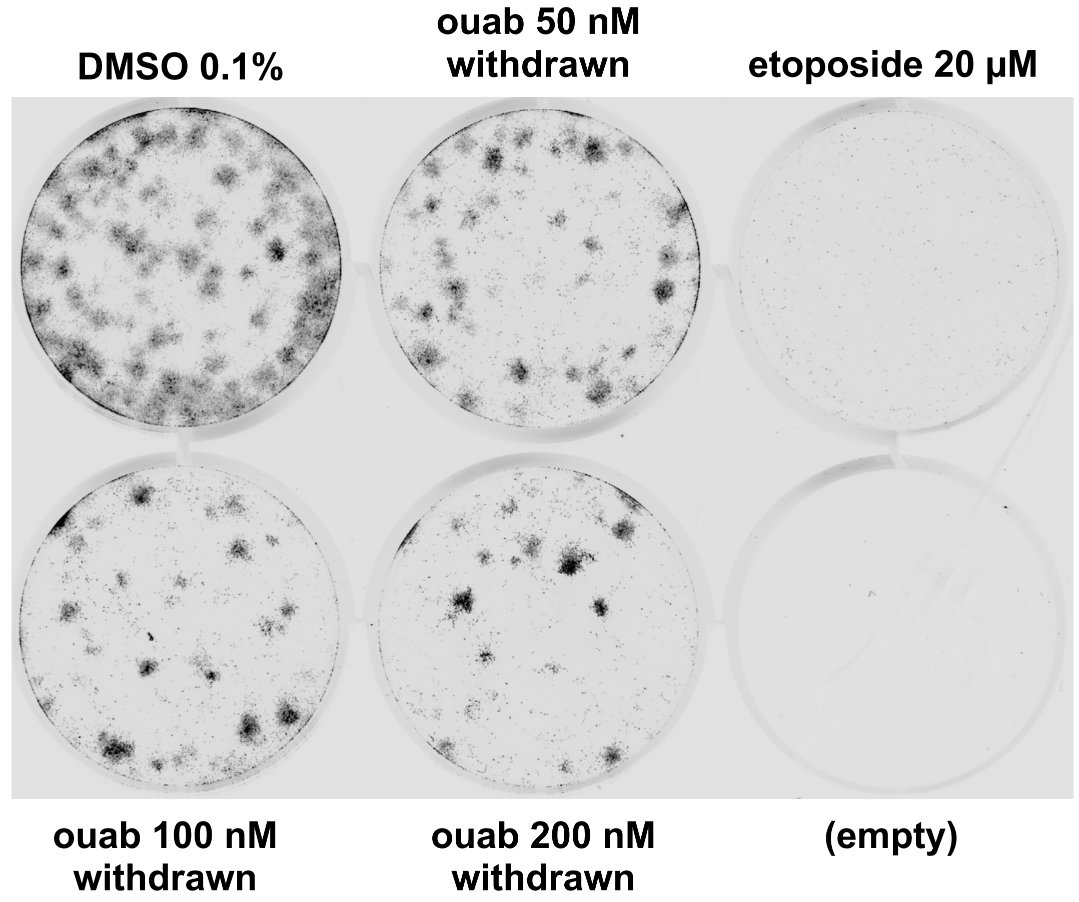

Figure S2. Ouabain-induced apoptosis hallmarks in BRafSen cells.
L'Hôte et al.

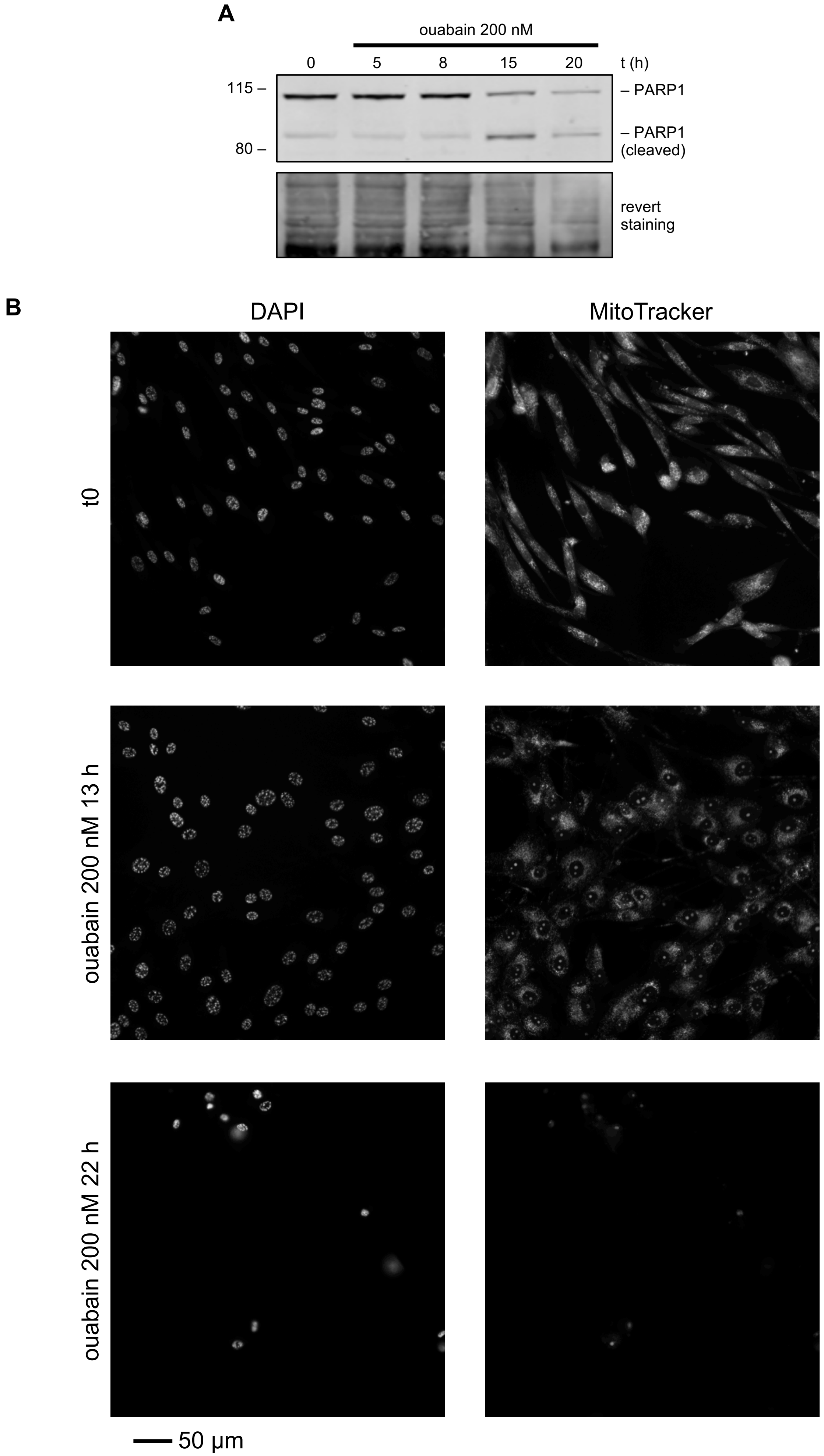

Figure S3. Cardioglycosides digoxin and strophanthidin exhibit the same toxicity profile as ouabain in BJ cells.
L'Hôte et al.

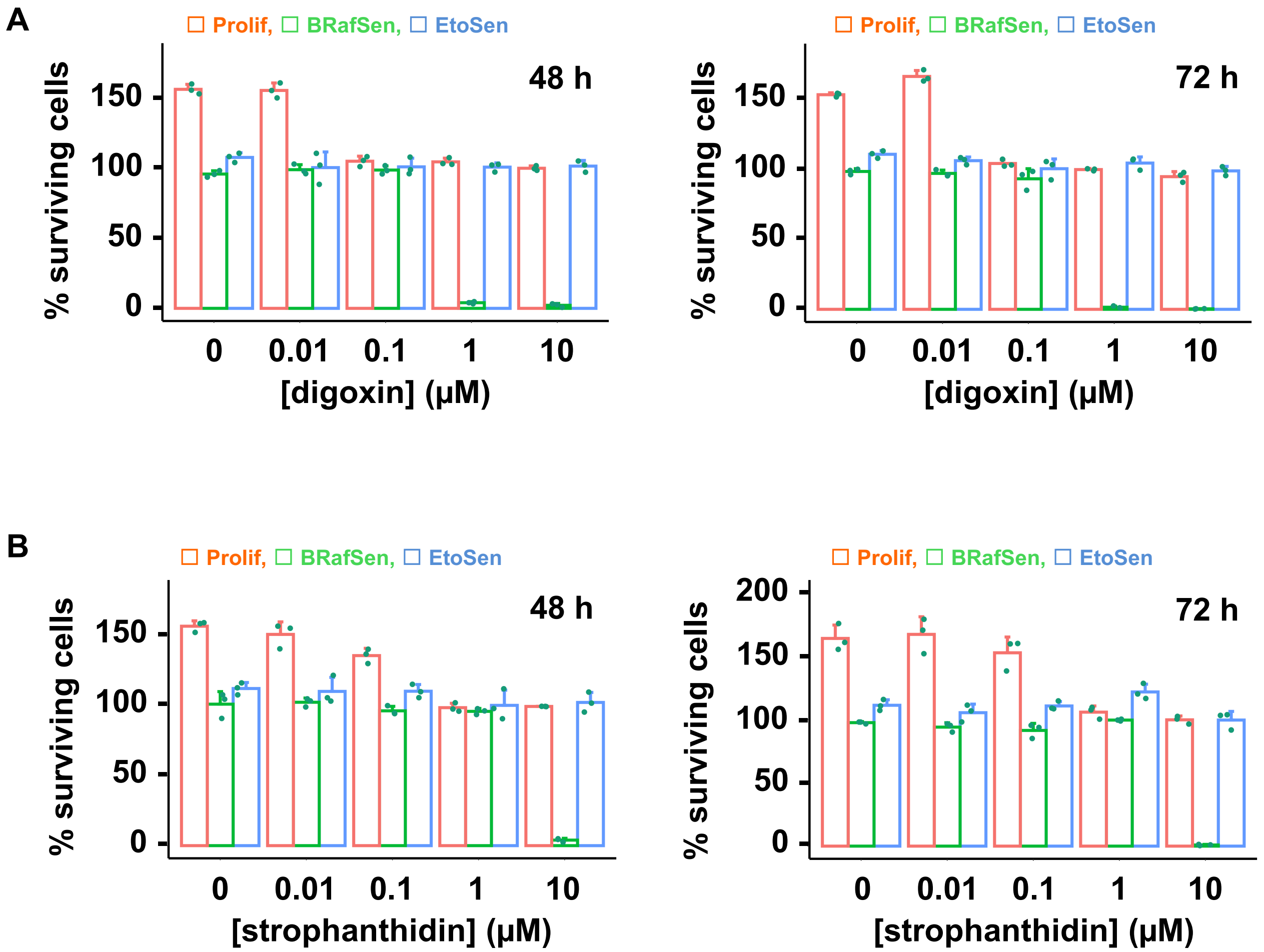

Figure S4. Senolytic activity of reference drugs in BJ cells.
L'Hôte et al.

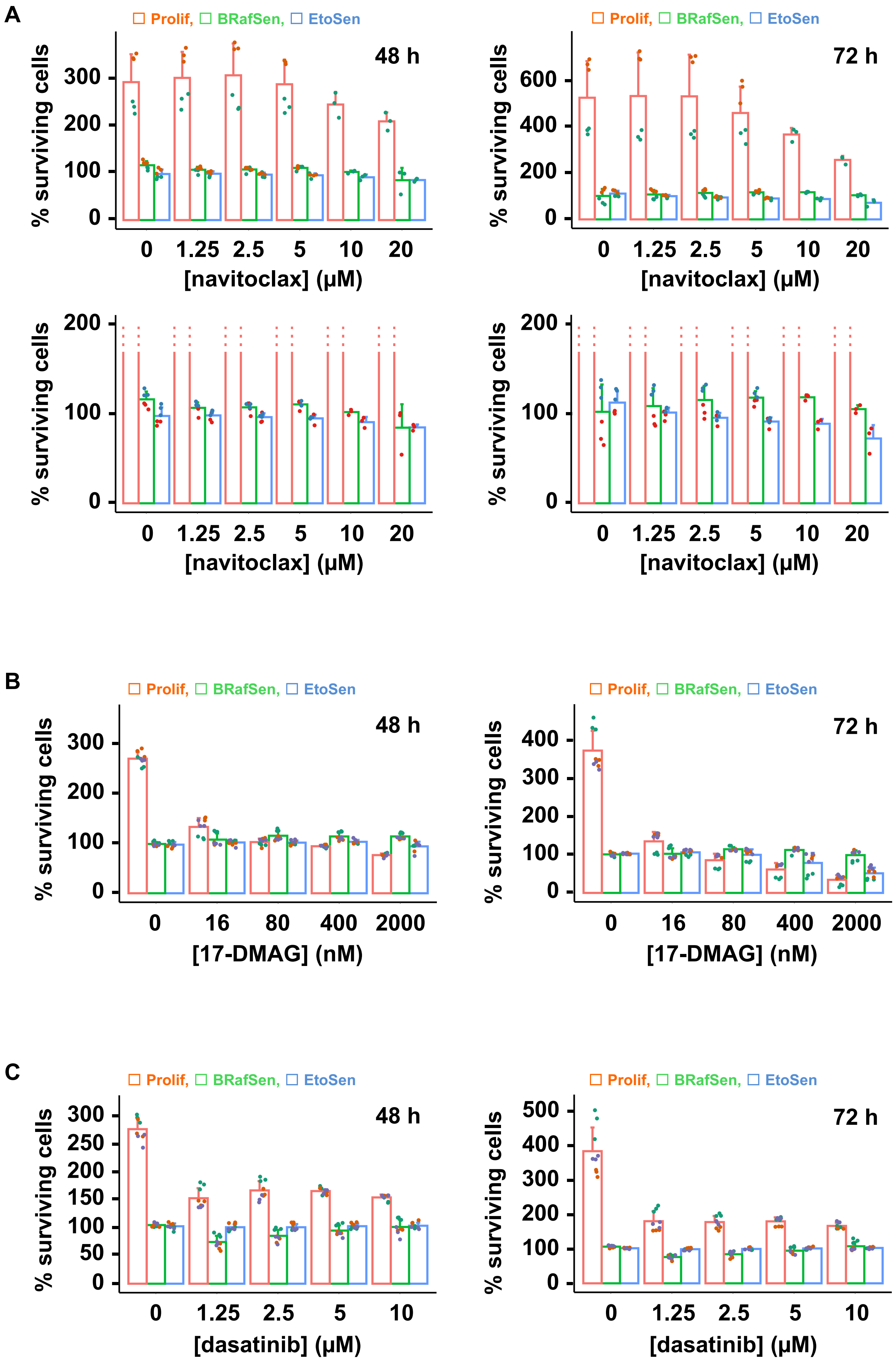

Figure S5. siRNA-mediated knockdown of ATP1A1.
L'Hôte et al.

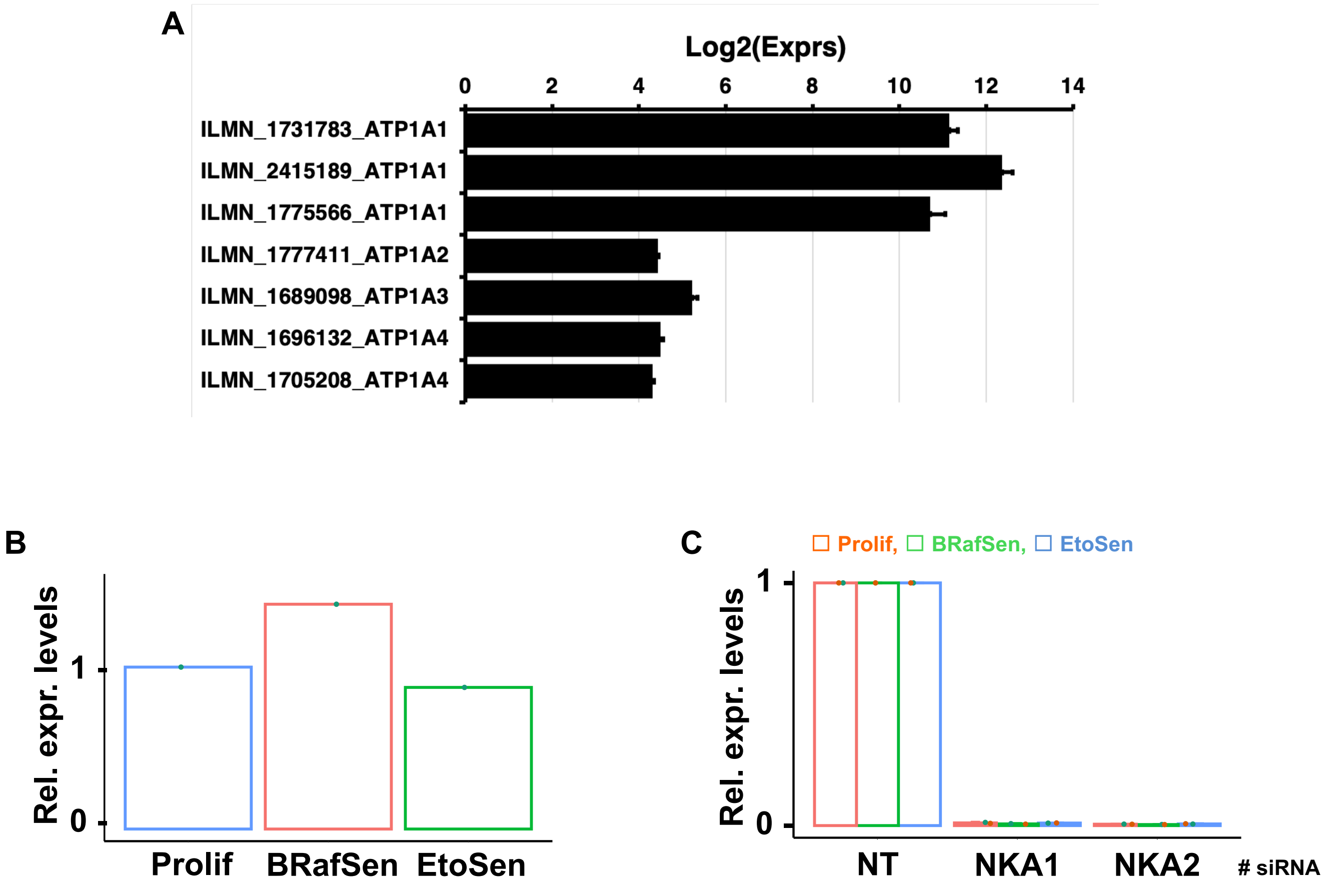

Figure S6. Total protein levels of Src, Erk, Akt, and p38, during ouabain and MB7 treatment, and ouabain and dasatinib treatment.
L'Hôte et al.

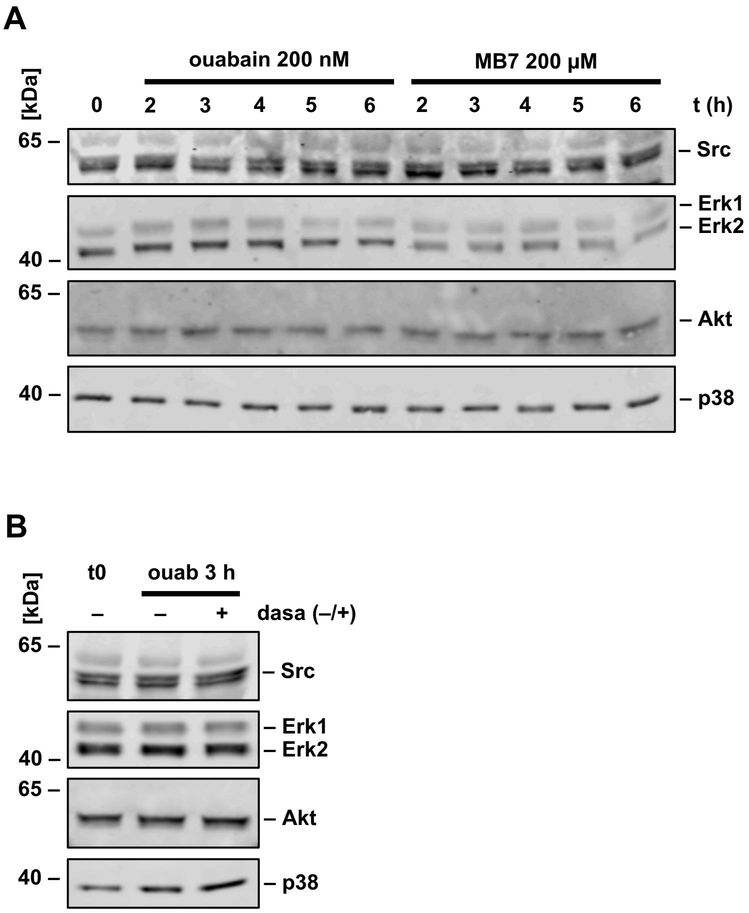

Figure S7. Ouabain differentiates between senescent and non-senescent cells overexpressing BRAFV600E, and kills BRafSen cells regardless of BRAFV600E expression level.
L'Hôte et al.

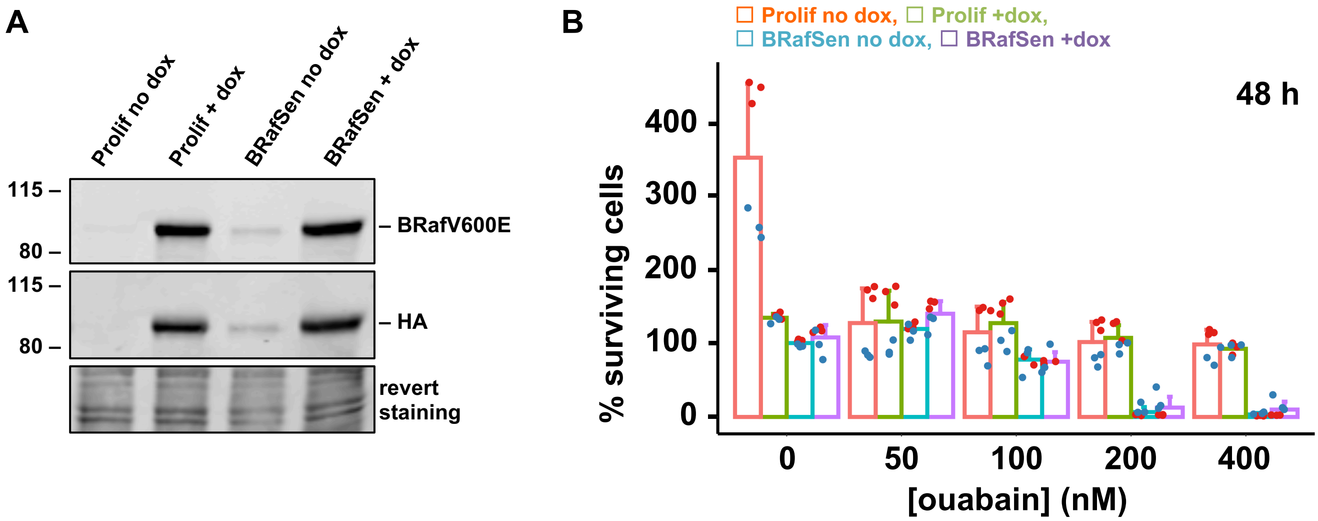

Figure S8. Autophagy inhibitor bafilomycin A1 is a senolytic in BRAF senescence.
L'Hôte et al.

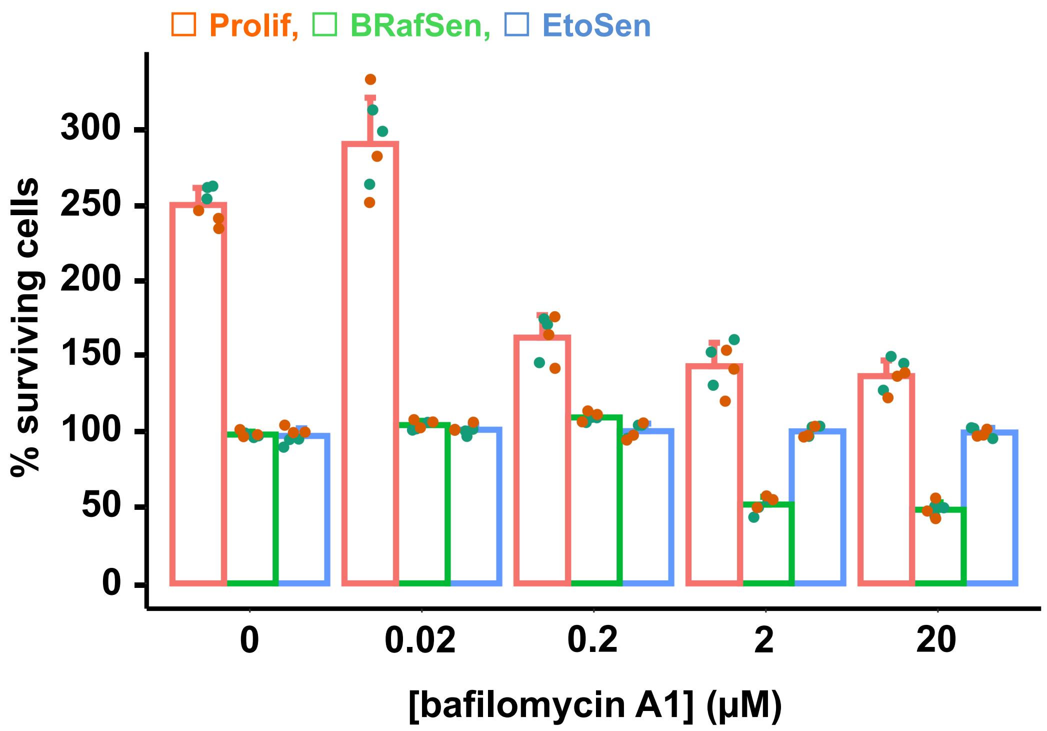

Figure S9. Cardioglycosides are broad-spectrum senolytics at higher concentrations.
L'Hôte et al.

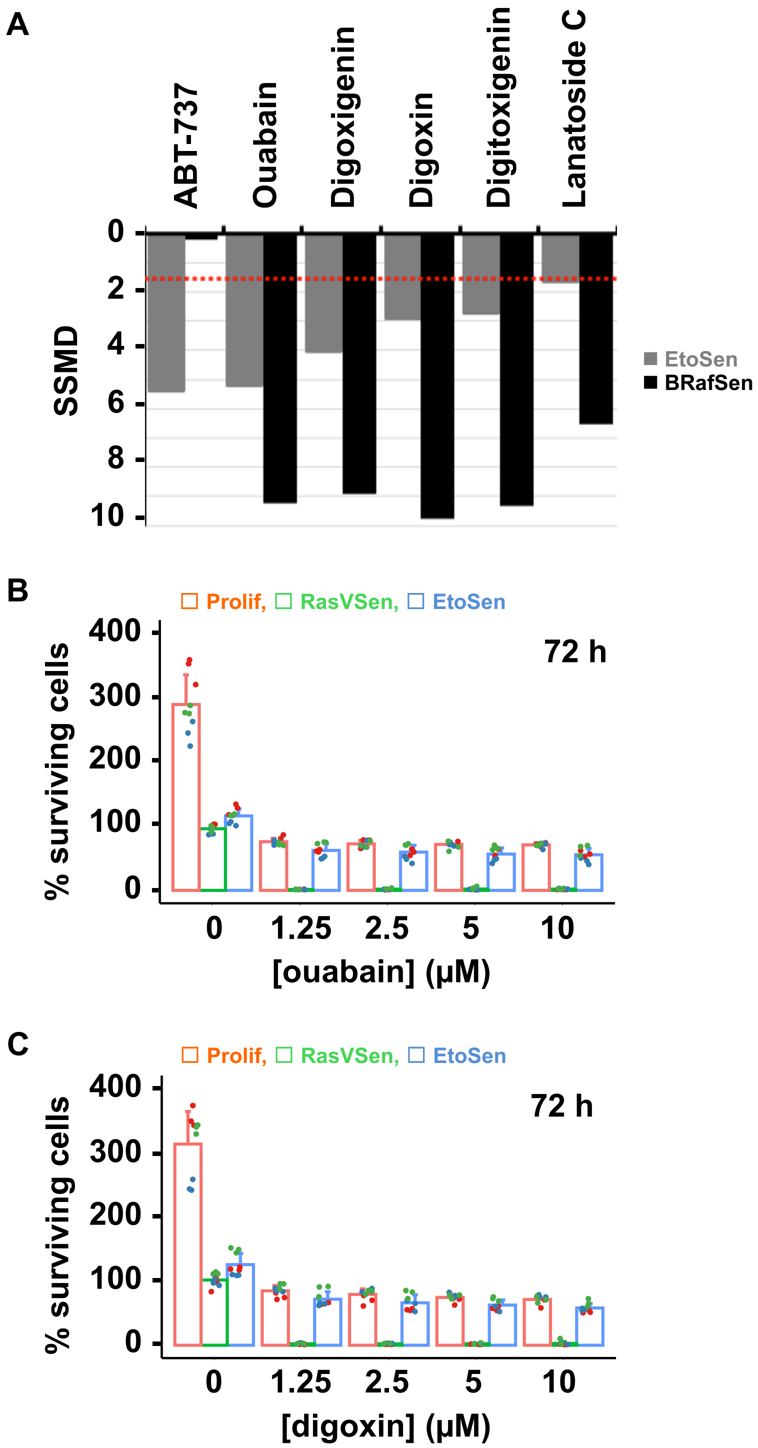

L'Hôte at al.

Supporting figure legends

Supporting Figure 1: Reversibility of ouabain-induced cytostaticity in proliferating cells.

A. EdU incorporation assay of Prolif BJ cells after ouabain withdrawal. We incubated Prolif cells with 50, 100, or 200 nM ouabain for 72 h. Then, we washed the wells with PBS and further incubated the cells with either the same concentration of ouabain or without ouabain, in the presence of 25 nM EdU for 48 h. We then fixed the cells and performed click-chemistry to measure the percentage of cells that carried out S phase (EdU-positive cells) during EdU incubation in various conditions. We used 20 µM etoposide as a negative control for proliferation. Representative experiment of two independent replicates.

B. Clonogenicity assay of Prolif cells after ouabain withdrawal. We incubated Prolif cells with 50, 100, or 200 nM ouabain for 72 h. Then, we washed and trypsinized the cells, and reseeded them at low density without ouabain. We used 20 µM etoposide as a negative control for proliferation. We cultured the cells for two weeks, renewing medium every 3 to 4 days, then fixed the cells and stained the colonies with crystal violet. Representative experiment of three independent replicates.

Supporting Figure 2: Ouabain-induced apoptosis hallmarks in BRAF-senescent cells.

A. PARP1 cleavage in BRafSen cells, as assessed by western blotting following incubation with 200 nM ouabain for various periods of time. Revert staining: total protein staining.

B. Fluorescence microscopy imaging of BRafSen cells treated with 200 nM ouabain for various periods of time, showing loss of mitochondrial potential (MitoTracker, 13 h), and nuclear condensation (DAPI, 22 h). Representative experiment of two independent replicates.

Supporting Figure 3: Cardioglycosides digoxin and strophanthidin exhibit the same toxicity profile as ouabain in BJ cells.

A. Dose-response toxicity assay of digoxin in BJ fibroblasts. Prolif: proliferating (red), BRafSen: BRAF-V600E senescent (green), EtoSen: etoposide senescent (blue). We expressed survival as the percentage of viable cells remaining attached to the well after incubation with the drug for 48 h (left) or 72 h (right), normalized to the initial number of cells at the time of drug addition.

B. Dose-response toxicity assay of strophanthidin in BJ fibroblasts.

For all panels, colored overlapping dots represent independent replicates.

Supporting Figure 4: Senolytic activity of reference drugs in BJ cells.

A. Dose-response toxicity assay of navitoclax / ABT-263 in BJ cells. Prolif: proliferating (red), BRafSen: BRAF-V600E senescent (green), EtoSen: etoposide senescent (blue). We expressed survival as the percentage of viable cells remaining attached to the well after incubation with the drug for 48 h (left) or 72 h (right), normalized to the initial number of cells at the time of drug addition. Zoomed-in graphs are provided in the lower row. Data were aggregated from two independent biological replicates.

B. Dose-response toxicity assay of alvespimycin / 17-DMAG in BJ cells. Data were aggregated from three independent biological replicates.

C. Dose-response toxicity assay of dasatinib in BJ cells. Data were aggregated from three independent biological replicates.

For all panels, colored overlapping dots represent independent replicates.

Supporting Figure 5: siRNA-mediated knockdown of ATP1A1 in BJ cells.

A. Transcriptomics analysis of Illumina beadarray data reveals that α1 is the most expressed α subunit isoform of the Na,K-ATPase pump in BRafSen BJ cells. The NKAα1 protein subunit is encoded by the ATP1A1 gene.

B. ATP1A1 mRNA levels in Prolif, BRafSen, and EtoSen cells, as measured by RT-qPCR. Cells had been induced in senescence for 7 days before RNA extraction.

C. ATP1A1 mRNA levels in BJ cells following transfection with ATP1A1-targeting siRNAs (si-NKA1 and si-NKA2) for 48 h, as measured by RT-qPCR. Virtually 100% of ATP1A1 mRNA levels are effectively depleted by both siRNAs in all cell lines. NT = no target control siRNA. Data were aggregated from two independent biological replicates.

Supporting Figure 6: Total protein levels of Src, Erk, Akt, and p38, during ouabain and MB7 treatment, and ouabain and dasatinib treatment.

A. As assessed by western blotting. Protein extracts are identical to those displayed in Figure 4A. Cells were treated with 200 nM ouabain or 200 µM MB7. Revert staining: total protein staining.

B. As assessed by western blotting. Protein extracts are identical to those displayed in Figure 4B. Cells were treated with 200 nM ouabain with or without 10 µM dasatinib for 3 h. Revert staining: total protein staining.

Supporting Figure 7: ouabain differentiates between senescent and non-senescent cells overexpressing BRAFV600E, and kills BRafSen cells regardless of BRAFV600E expression level.

A. Protein levels of BRafV600E/HA in proliferating and 1-week BRafSen cells seeded 24 h before with (+dox) or without (no dox) 1 µg/mL doxycycline, as assessed by western blotting. Revert staining: total protein staining. Representative experiment of four independent replicates.

B. Dose-response toxicity assay of ouabain in proliferating and 1-week BRafSen cells seeded 24 h before drug addition with (+dox) or without (no dox) 1 µg/mL doxycycline. The cells were treated with ouabain for 48 h. Prolif: proliferating (red), BRafSen: BRAF-V600E senescent (green). We expressed survival as the percentage of viable cells remaining attached to the well after incubation with the drug for 48 h normalized to the initial number of cells at the time of drug addition. Data were aggregated from two independent biological replicates. Colored overlapping dots represent independent replicates.

Supporting Figure 8: Autophagy inhibitor bafilomycin A1 is a senolytic in BRAF senescence.

Dose-response toxicity assay of bafilomycin A1 in BJ cells. Prolif: proliferating (red), BRafSen: BRAF-V600E senescent (green), EtoSen: etoposide senescent (blue). We expressed survival as the percentage of viable cells remaining attached to the well after incubation with the drug for 48 h, normalized to the initial number of cells at the time of drug addition. Data were aggregated from two independent biological replicates. Colored overlapping dots represent independent replicates.

Supporting Figure 9: Cardioglycosides are broad-spectrum senolytics at higher concentrations.

A. Cardioglycosides as senolytic screening hits of the Prestwick library (ouabain: supplementary control) in both BRafSen and EtoSen BJ cells. Cells were incubated with drugs at 20 µM for 72 h. Surviving cell count was determined after fixation and nuclei staining using

a CX5 screening microscope. Strictly standardized mean difference (SSMD) was calculated for each compound, and a score of SSMD<1.645 (red dotted line) was considered a hit.

B. Dose-response toxicity assay of ouabain in IMR90 cells. Prolif: proliferating (red), RasVSen: RasVal12 senescent (green), EtoSen: etoposide senescent (blue). We expressed survival as the percentage of viable cells remaining attached to the well after incubation with the drug for 72 h, normalized to the initial number of cells at the time of drug addition. Data were aggregated from three independent biological replicates.

C. Dose-response toxicity assay of digoxin in IMR90 cells. Data were aggregated from three independent biological replicates.

For all panels, colored overlapping dots represent independent replicates.
